# Nonalcoholic Fatty Liver Disease Exacerbates the Advancement of Renal Fibrosis by Modulating Renal CCR2^+^PIRB^+^ Macrophages Through the ANGPTL8/PIRB/ALOX5AP Axis

**DOI:** 10.1002/advs.202509351

**Published:** 2025-09-25

**Authors:** Shuqi Wei, Diwen Shou, Siyuan Huang, Yingsi Wang, Quanzhen Liu, Jinfa Liu, Xiaoyu Lv, Yuyi Zeng, Liting Wei, Han Lin, Jiechuan Chen, Yangxiao Chen, Haoyu Zhong, Yanqing Zhou, Wen Ma, Li Wang, Guojun Qian, Jing Guo, Donglin Sun

**Affiliations:** ^1^ Department of Urology Shenzhen Hospital Southern Medical University Shenzhen Guangdong 518100 China; ^2^ The Third Affiliated Hospital of Sun Yat‐sen University Guangzhou 510630 China; ^3^ Center for Cancer and Immunology Research State Key Laboratory of Respiratory Disease Affiliated Cancer Hospital of Guangzhou Medical University Guangzhou 510182 China; ^4^ Department of Anesthesiology Guangdong Provincial Key Laboratory of Major Obstetric Diseases Guangdong Provincial Clinical Research Center for Obstetrics and Gynecology The Third Affiliated Hospital Guangzhou Medical University Guangzhou 510150 China; ^5^ Department of Gastroenterology and Hepatology Guangzhou Digestive Disease Center Guangzhou First People's Hospital Guangzhou 510180 China; ^6^ Department of Gastroenterology and Hepatology the Second Affiliated Hospital School of Medicine South China University of Technology Guangzhou 510180 China; ^7^ Department of Gynecology and Obstetrics The First Affiliated Hospital of Guangzhou Medical University Guangzhou Guangdong 510120 China; ^8^ Department of Gynecology and Obstetrics General Hospital of Southern Theater Command Guangzhou Guangdong 510010 China; ^9^ Guangzhou Medical University Guangzhou Guangdong 510030 China; ^10^ Department of Physiology and Pathophysiology School of Basic Medical Sciences Fudan University Shanghai 200032 China; ^11^ College of Intelligent Medicine and Biotechnology Lingui Campus Guilin Medical University Guilin 541199 China; ^12^ The Clinical Innovation & Research Center (CIRC) Shenzhen Hospital Southern Medical University Shenzhen Guangdong 518100 China; ^13^ Clinical Laboratory Shenzhen Hospital Southern Medical University Shenzhen Guangdong 518100 China; ^14^ Southern Medical University Affiliated Longhua People's Hospital Nephrology department Shenzhen Guangdong 518000 China; ^15^ Key Laboratory of Anesthesiology Shanghai Jiao Tong University Ministry of Education Ren Ji Hospital Shanghai 200127 China; ^16^ Center of oncology Heyou Hospital Shunde District Foshan City Guangdong 528000 China

**Keywords:** ALOX5AP, ANGPTL8, macrophages, nonalcoholic fatty liver disease, renal fibrosis

## Abstract

Renal fibrosis is a critical pathological hallmark of chronic kidney disease. Although nonalcoholic fatty liver disease (NAFLD) has been implicated in kidney disease progression, its specific role and underlying mechanisms in renal fibrosis remain poorly understood. This study aims to investigate these mechanisms by establishing a mouse model of renal fibrosis through unilateral ureteral obstruction (UUO) combined with a high‐fat diet‐induced NAFLD. Single‐cell RNA sequencing, untargeted metabolomics, flow cytometry, and immunofluorescence are performed, along with in vitro experiments involving primary renal macrophages and coculture models. It is demonstrated that NAFLD exacerbates renal fibrosis, as HFD‐induced hepatocytes release significant levels of ANGPTL8, which activates renal CCR2^+^PIRB^+^ macrophages. These specialized macrophages disrupt linoleic acid metabolism and increase the production of inflammatory cytokines, aggravating renal fibrosis. In addition, CCR2^+^PIRB^+^ macrophages promote the activation and proliferation of Th17 cells, which can further contribute to the worsening of renal fibrosis. Thus, the ANGPTL8/PIRB/ALOX5AP axis is a crucial signaling pathway between the liver and kidneys, and CCR2^+^PIRB^+^ macrophages play a pivotal role in the progression of NAFLD‐induced renal fibrosis. These findings suggest potential therapeutic targets to treat NAFLD‐related renal fibrosis.

## Graphical abstract

The ANGPTL8/PIRB/ALOX5AP axis mediates liver‐kidney crosstalk. Hepatocytes secrete ANGPTL8, which binds to CCR2^+^PIRB^+^ macrophages in the kidneys, affecting the expression of ALOX5AP and enhancing the activation of these macrophages. Activated cells produce TGF‐β, IL‐6, and IL‐23, driving Th17 activation and promote the formation of myofibroblasts, while CCR2^+^PIRB‐ macrophages secrete PDGFB promoting fibroblast proliferation, leading to the exacerbation of renal fibrosis. Created in BioRender. (2025) https://BioRender.com/g3s0otc

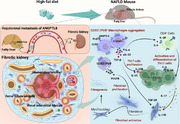

## 1 Introduction

Chronic kidney disease (CKD) is characterized by structural and functional impairment of the kidneys resulting from various causes and is characterized by a low glomerular filtration rate (GFR) and persistent kidney injury indicators for at least three months.^[^ ^1^ ^]^ Kidney fibrosis, a common pathological feature of CKD and a precursor of end‐stage renal failure, significantly threatens the quality of life of patients.^[^ ^2^ ^]^ Fibrosis arises from repeated cycles of injury and repair, in which immune cells and transformed myofibroblasts secrete proinflammatory and profibrotic cytokines, respectively, leading to excessive deposition of extracellular matrix (ECM) and tissue scarring.^[^ ^3^ ^]^ However, no treatments have been developed to reverse renal fibrosis. Current drugs in clinical trials have shown limited efficacy and considerable side effects^[^ ^4^, ^5^ ^]^ indicating that our understanding of the mechanisms underlying kidney fibrosis and the development of individualized therapies remains insufficient. Obesity is a major risk factor for CKD progression.^[^ ^6^ ^]^ Moreover, it has been established that obesity contributes to the development of NAFLD^[^ ^7^, ^8^ ^]^. Therefore, NAFLD may play a crucial role in renal fibrosis, and the potential complex interactions between liver and kidney functions remain to be fully elucidated.^[^ ^9^, ^10^ ^]^ We hypothesize that aberrant activation of the liver‐kidney axis and adipose‐kidney axes may be critical in this process, necessitating further in‐depth investigation.^[^ ^11^ ^]^ NAFLD is characterized by abnormal hepatic fat accumulation, which may induce the release of proinflammatory lipid mediators and lipid transport proteins into the systemic circulation,^[^ ^12^ ^]^ leading to lipotoxicity, inflammation, oxidative stress, and fibrosis, which are closely linked to the progression of CKD.^[^ ^13^, ^14^ ^]^ Existing research on the liver‐kidney axis has notable limitations: on one hand,^[^ ^15^ ^]^ previous studies have primarily focused on the hemodynamic abnormalities of hepatorenal syndrome or the direct tubular damage caused by lipotoxicity,^[^ ^16^ ^]^ overlooking the pivotal role of the immune system in inter‐organ communication; however, investigations into the inflammatory mechanisms of NAFLD have not been integrated into the mechanisms of CKD, ^[^ ^17^ ^]^ and how the liver reshapes the renal immune microenvironment through endocrine signals remains unexplored in current research.^[^ ^18^, ^19^ ^]^

Angiopoietin‐like protein 8 (ANGPTL8) is a unique cytokine within the angiopoietin‐like family that lacks the typical coiled‐coil domain and is exclusively expressed in the human liver.^[^ ^20^, ^21^ ^]^ Elevated levels of ANGPTL8 have been observed in both mouse models of NAFLD and human patients.^[^ ^22^ ^]^ Studies have reported that high ANGPTL8 levels interact with paired immunoglobulin‐like receptor B (PIRB), an immunosuppressive receptor expressed on liver macrophages, activating NF‐κB and accelerating NAFLD progression.^[^ ^23^ ^]^ Similarly, macrophages are the most prominent immune cells within the renal tissue, are key effectors of renal inflammation^[^ ^24^, ^25^ ^]^ and play crucial roles in both kidney injury and repair.^[^ ^26^ ^]^ Among these signaling pathways, the CCR2‐CCL2 receptor‐ligand axis is essential for macrophage chemotaxis and the progression of inflammation.^[^ ^25^, ^27^ ^]^ Following UUO surgery, circulating monocytes are specifically recruited to the obstructed kidney in the early phase, subsequently expressing the macrophage surface marker F4/80 and maintaining CCR2 expression. CCR2^+^ macrophages continue to respond to damage‐associated molecular patterns expressed by injured cells, contributing to the development of renal fibrosis.^[^ ^28^ ^]^ Linoleic acid, an ω‐6 polyunsaturated fatty acid, when present in excess, promotes the metabolism of related enzymes and consequently induces chronic inflammation.^[^ ^29^ ^]^ The accumulation of linoleic acid in the kidneys is significant, as it profoundly affects the progression of renal fibrosis.^[^ ^30^, ^31^ ^]^ Recent studies have also indicated that linoleic acid exhibits a high affinity for macrophages, promoting inflammatory progression. Lipoxygenases (LOXs), which are expressed in immune cells as the initial catalysts for linoleic acid oxidation, can lead to the formation of lipid mediators that activate various signaling pathways.^[^ ^32^ ^]^ Among the LOX subtypes, LOX‐5, encoded by ALOX5, has been identified as a key enzyme in linoleic acid metabolism.^[^ ^33^, ^34^, ^35^ ^]^ The activity of LOX‐5 is contingent on its interaction with 5‐lipoxygenase‐activating protein (FLAP), which is encoded by ALOX5AP and located on the nuclear membrane.^[^ ^33^ ^]^

However, the following scientific questions remain unresolved: Does NAFLD‐derived ANGPTL8 specifically target distal renal macrophages? Does this process define an NAFLD‐specific macrophage subset? What downstream signals convert the liver‐kidney axis signal into a profibrotic signal?

Given the significant correlation between NAFLD and renal fibrosis, we propose the central hypothesis that ANGPTL8 derived from NAFLD activates the ALOX5AP pathway in renal CCR2^+^PIRB^+^ macrophages via PIRB, reprogramming linoleic acid metabolism, and driving profibrotic inflammation. In this study, we established NAFLD‐ and UUO‐induced renal‐fibrosis models, used single‐cell transcriptomic sequencing and untargeted metabolomic sequencing, and identified the ANGPTL8/PIRB/ALOX5AP axis as a novel hub linking hepatic lipotoxic damage to renal immune‐metabolic dysregulation. In this communication axis, fatty liver cells secrete ANGPTL8, which activates CCR2^+^PIRB^+^ macrophages in the kidneys. By influencing the linoleic acid metabolism of this specific cell subset, it promotes the production of cytokines such as IL‐6, IL‐23, and TGF‐β, which exacerbate renal fibrosis, thus enabling the liver to regulate the kidneys. Targeting ALOX5AP can block this inter‐organ axis and alleviate fibrosis. Our study provides a new strategy for investigating immune‐metabolic interactions in NAFLD‐related renal fibrosis, laying the foundation for the development of targeted therapies.

## 2 Results

### 2.1 Mendelian Randomization Combined with Pathological Phenotyping Confirms that NAFLD Exacerbates Renal Fibrosis in CKD

**Figure 1:**
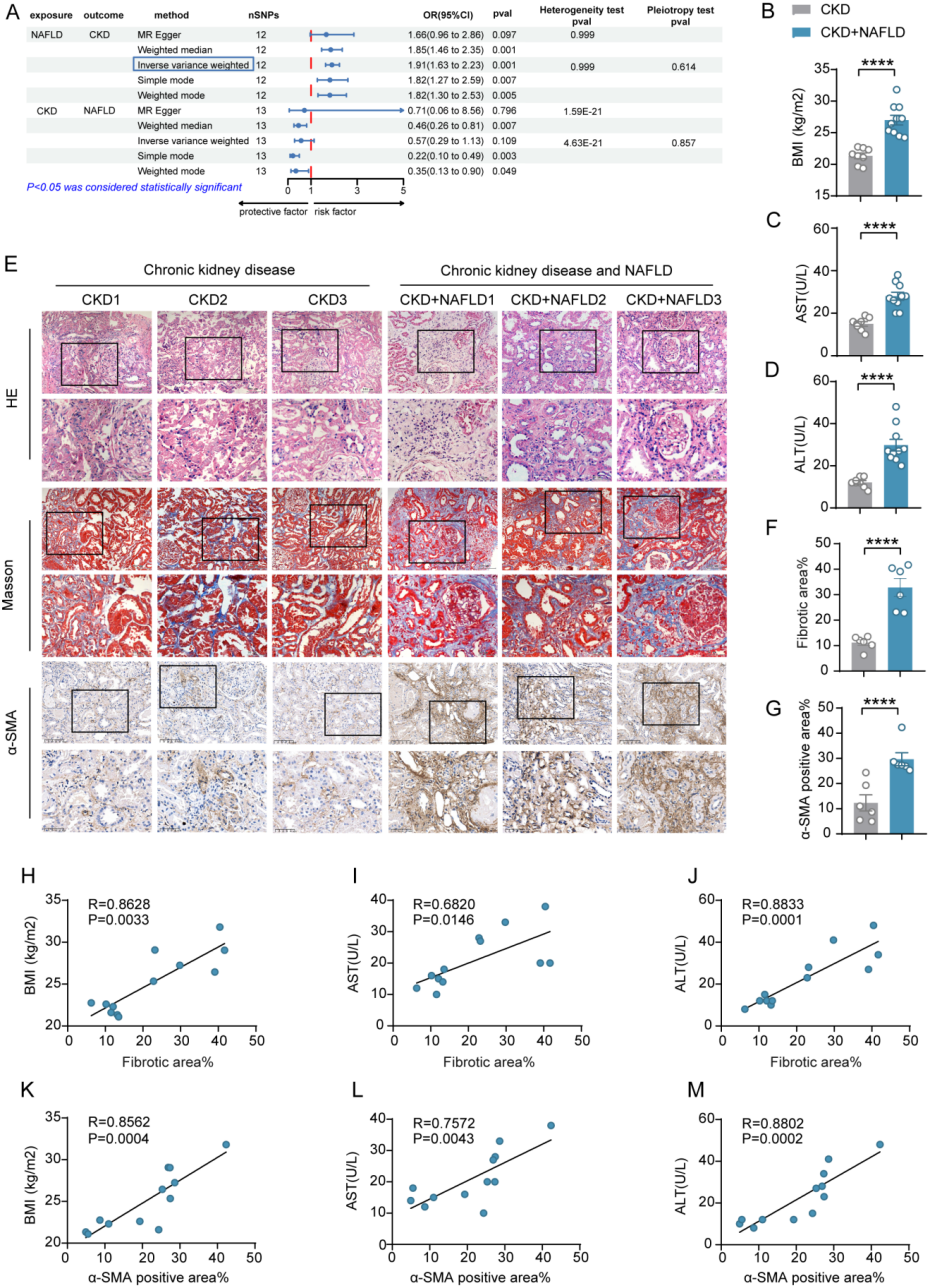
NAFLD exacerbates renal fibrosis in patients with CKD.A) Forest plot showing the association between NAFLD and CKD risk. NAFLD, NAFLD; CKD, chronic kidney disease; SNPs, single nucleotide polymorphisms; OR, odds ratio; CI, confidence interval.B) Quantitative analysis of the body mass index (calculated as W/H^2^). C,D) Serum levels of transaminases AST and ALT. E) Histopathology of renal fine‐needle aspiration biopsies from patients with CKD and those with CKD and comorbid NAFLD. Representative images: Hematoxylin & eosin (H&E): Tissue architecture, Masson's trichrome: Collagen deposition (blue), α‐SMA immunohistochemistry (IHC): Myofibroblast activation (brown), Scale bar: 100 µm, 50 µm.F) Quantitative analysis of collagen deposition (Masson's trichrome‐positive area).G) Quantification of α‐SMA⁺ from IHC staining. H‐J, Correlation analysis between Masson‐positive areas and BMI, AST, and ALT levels. K‐M, Correlation analysis between α‐SMA positive area and BMI, AST, ALT levels. B─D, F─G) The data are presented as the means ± SEM (n = 6‐10). Group comparisons were analyzed using a two‐tailed Student's *t*‐test;*p < 0.05, **p < 0.01, ***p < 0.001, ****p < 0.0001. H‐M: Correlation analyses were conducted using Pearson's correlation coefficient.

First, we obtained NAFLD patient data from the GWAS Catalog database (GCST90091033) and CKD patient data from the IEU OpenGWAS project website. The primary analysis was conducted using the inverse variance‐weighted (IVW) method, supplemented by the weighted median estimator (WME), MR‐Egger regression, simple models, and weighted models, with SNPs as instrumental variables to analyze the causal relationship between NAFLD and CKD. A direct causal relationship has been identified between NAFLD and CKD, suggesting that NAFLD may increase the risk of developing renal fibrosis. However, reverse MR analysis did not reveal a significant association between CKD and NAFLD (*p >* 0.05), indicating that there is no evidence to support the notion that renal fibrosis could lead to NAFLD (**Figures** **1**A, S1A–H, Supporting Information). Subsequently, we selected a cohort of patients with CKD with biopsy‐confirmed renal fibrosis and divided them into two groups based on the presence or absence of NAFLD: those with only renal fibrosis (CKD) and those with both renal fibrosis and NAFLD (CKD + NAFLD). The CKD + NAFLD group exhibited significantly higher BMI, AST, and ALT levels than the CKD group (Figure 1B–D). Kidney biopsy samples from these patients were subjected to hematoxylin and eosin (HE) staining, Masson's trichrome staining, and immunohistochemical staining for α‐SMA (Figure 1E). Compared to the CKD group, the CKD+NAFLD group had markedly increased vacuolar dilation of renal tubular epithelial cells and a significant increase in blue deposits positive for Masson staining (Figure 1F) and a notable increase in brown α‐SMA immunohistochemical positivity (Figure 1G), which correlated positively with BMI, AST, and ALT levels (Figure 1H–M).

Our Mendelian randomization analysis and patient tissue morphology staining methods revealed that patients with CKD and fatty liver disease exhibited more pronounced renal fibrosis.

### 2.2 The Severity of Renal Fibrosis in NAFLD Mice is Heightened Following UUO

**Figure 2:**
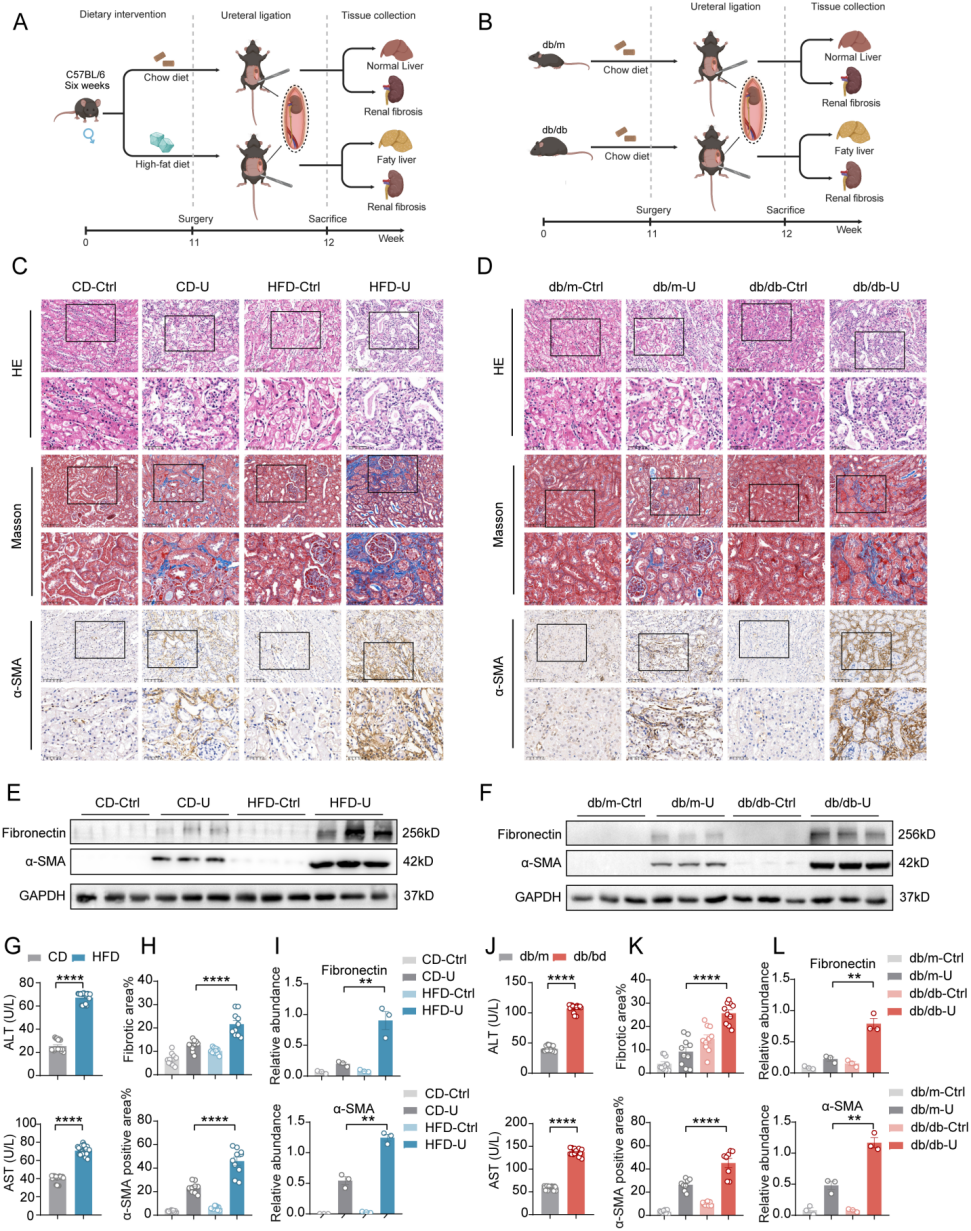
Increased renal fibrosis severity in NAFLD mice following UUO. A) Experimental workflow for high‐fat diet‐induced NAFLD with renal fibrosis: UUO was performed after 11 weeks of high‐fat diet or normal chow feeding. After 7 days, the livers and kidneys were collected for evaluation. B) Experimental workflow for db/db mouse model of NAFLD and renal fibrosis: Age‐matched db/m and db/db mice were fed a normal chow diet. UUO surgery was performed at 11 weeks of age. After 7 days, the livers and kidneys were collected for evaluation. C) High‐fat diet (HFD) and control diet (CD) mice: UUO surgery was performed on both right (nonligated, HFD‐Ctrl, CD‐Ctrl) and left (ligated, HFD‐U, CD‐U) kidneys 7 days post‐surgery. HE staining was used to show renal tissue morphology, Masson's staining indicated collagen deposition (blue), and immunohistochemistry was used to display α‐SMA expression (brown). (Scale bar: 100 µm.) D) db/db, and db/m mice: UUO surgery was performed on both the right (nonligated, db/db‐Ctrl, db/m‐Ctrl) and left (ligated, db/db‐U, db/m‐U) kidneys 7 days post‐surgery. HE staining displayed renal tissue morphology, Masson's staining highlighted collagen deposition (blue), and immunohistochemistry showed α‐SMA expression (brown). (Scale bar: 100 µm.) Scale bar: 100 µm. E,F) Western blot analysis of fibrosis‐related protein expression in kidneys. G) Plasma ALT and AST levels in HFD and CD mice. H) Quantification of Masson's staining and α‐SMA immunohistochemistry in HFD and CD mice. I) Quantification of Western blot results for HFD and CD mice. J. Plasma ALT and AST levels in db/db and db/m mice (*n* = 3). K) Quantification of Masson's staining and α‐SMA immunohistochemistry in db/db and db/m mice. L) Quantification of Western blot results for db/db and db/m mice (*n* = 3). G─L) The data are presented as the means ± SEM (*n* = 10). Group comparisons were analyzed using a two‐tailed Student's *t*‐test; **p < 0.01, ***p < 0.001, ****p < 0.0001.

To assess whether NAFLD exacerbates fibrosis after UUO, we selected two mouse models: a high‐fat diet model and a db/db model. First, C57BL/6 mice were fed a high‐fat diet (HFD) or standard diet (CD) for 12 weeks to establish the NAFLD model, and the control group was fed a standard diet (**Figure** **2**A). The db/db and control db/m mice were fed a standard diet for 12 weeks. Owing to a mutation in the leptin gene, db/db mice developed NAFLD (Figure 2B). Subsequently, in the 11th week, we subjected both the NAFLD models and their corresponding control groups to UUO surgery to establish a renal‐fibrosis model. After seven days, blood, liver, and kidney samples were collected from the mice for evaluation. Both the db/db and HFD groups exhibited significant weight gain (Figure S2C,D, Supporting Information). Macroscopic observations revealed enlarged livers with a pale color in the HFD group (Figure S2A, Supporting Information), and the same phenomenon was observed in both db/m and db/db mice. HE staining revealed an orderly liver cell arrangement and uniform cytoplasm in the CD and db/m groups. In contrast, the HFD and db/db groups exhibited numerous lipid droplets of varying sizes in the cytoplasm, indicating ballooning degeneration. Oil Red O staining revealed distinct red fat droplets in the livers of HFD and db/db mice, with an increased area and intensity of staining compared to those in the CD and db/m groups, indicating significant lipid accumulation (Figure S2E,F, Supporting Information). Liver function markers, such as ALT and AST, and blood lipid markers, including total cholesterol (TC) and triglyceride (TG) levels, were significantly elevated in the HFD and db/db groups compared to those in the CD and db/m groups (Figure 2G,J) (Figure S2G,H), confirming the successful establishment of the NAFLD model.

Seven days after establishing the UUO model, the kidneys of HFD UUO mice appeared significantly paler than those of normal diet UUO mice (Figure S2B, Supporting Information), and the same observations were made in both db/m and db/db mice. Histological analysis of kidney sections via HE staining revealed a normal glomerular structure and orderly arrangement of renal tubular epithelial cells in the kidneys of the nonligated side. In contrast, UUO kidneys of control group mice (CD‐U and db/m‐U) exhibited atrophic or necrotic renal tubules, some with dilatation and vacuolar changes, glomerulosclerosis, and increased renal interstitial fibrosis. The UUO kidneys of the NAFLD group mice (HFD‐U and db/db‐U) displayed more severe renal fibrosis than those of the control group (Figure 2C,D). Masson's staining indicated that the HFD‐U and db/db‐U groups had a larger area of renal tissue fibrosis than the CD‐U and db/m‐U groups (*p <* 0.05), which was predominantly distributed in the renal interstitium. Immunohistochemical results showed that α‐SMA staining in the HFD‐U and db/db‐U groups had a larger area and darker color, reflecting a significant increase in α‐SMA expression (*p <* 0.05) (Figure 2H,K). Western blot analysis confirmed that the expression levels of fibrosis markers, Fibronectin and α‐SMA, were significantly higher in the HFD‐U and db/db‐U groups than in the CD‐U and db/m‐U groups (*p <* 0.05) (Figure 2E,F,I,L). Furthermore, at the mRNA level, genes associated with fibrosis, such as Fibronectin, Collagen1, and α‐SMA, were significantly upregulated (Figure S2I,J, Supporting Information).

These findings suggest that in the NAFLD model, the severity of kidney fibrosis in the kidneys of the UUO model mice increased, with enhanced expression of major fibrosis markers.

### 2.3 The Significant Changes in the Number and Metabolic Function of CCR2^+^ Macrophages

**Figure 3:**
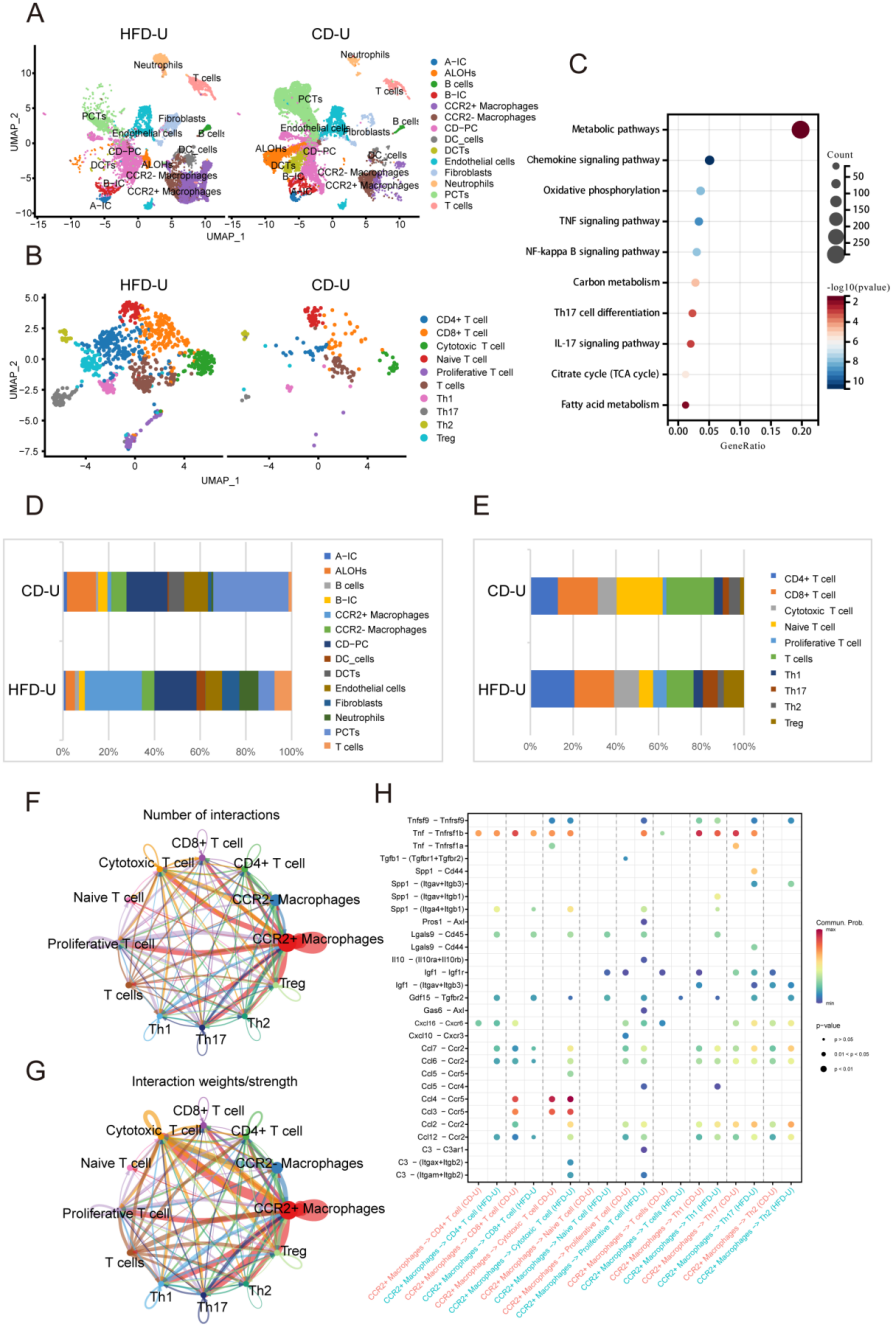
Single‐cell atlas of NAFLD and normal mouse kidney fibrosis. A) Uniform manifold approximation and projection (UMAP) dimension reduction of cells in UUO kidneys, showing 14 cell types identified by unsupervised clustering. CD‐PC, collecting duct principal cells; PCTs, proximal tubular cells; ALOHs, Ascending loop of Henle cells; DCTs, distal tubular cells; B‐IC, beta‐intercalated cells; A‐IC, alpha‐intercalated cells; Fibroblasts; Endothelial cells; CCR2^‐^ Macrophages; CCR2^+^ macrophages; neutrophils; DC_cells; B cells; T cells. B) Subclustering analysis of T cells in UUO kidneys. CD4^+^ T cells, CD8^+^ T cells, cytotoxic T cells, naïve T cells, proliferative T cells, Th1, Th17, Th2, and Tregs. C) KEGG analysis of differentially expressed genes in HFD‐U and CD‐U. D, Proportions of 14 cell type subpopulations. E) Proportion of T‐cell subsets. F) Differential diagram of cell interactions compared to CD‐U, with red edges indicating an increase in the number of cell interactions in HFD‐U mice and blue edges indicating a decrease. G) Differential diagram of cell‐interaction strength compared to CD‐U, with red edges indicating an increase in cell‐interaction strength in HFD‐U, and blue edges indicating a decrease. H) Differential analysis of signal ligand‐receptor pairs between CCR2^+^Macrophages and other cells in the CD‐U and HFD‐U groups.

We performed single‐cell sequencing of the kidneys of the HFD‐U and CD‐U groups, annotating 14 types of renal cells in the Uniform Manifold Approximation and Projection (UMAP) plots across the different model groups (**Figures** **3**A and S3A, Supporting Information). Subsequently, we quantified the number and proportion of each cell subpopulation, revealing a significant increase in the abundance of macrophages in the HFD‐U group compared to that in the CD‐U group (Figure 3D). Furthermore, we stratified the macrophages into two subtypes, CCR2^+^ and CCR2^‐^ (Figure S3B–D), based on the varying expression levels of the CCR2 receptor, which has been implicated as a discriminator between migratory/inflammation‐induced macrophages (CCR2^+^) and resident macrophages (CCR2^‐^).^[^ ^27^ ^]^ Moreover, we conducted a subpopulation analysis specifically focusing on T cells, annotating the UMAP plots to discern 10 discrete T‐cell subpopulations (Figure 3B). Subsequently, we quantified the number and proportion of each T‐cell subpopulation, revealing a significant increase in the abundance of Th17 and Treg subpopulations in the HFD‐U group compared to that in the CD‐U group (Figure 3E).

Monocle2 was used for cell sorting and the construction of a pseudotime trajectory to elucidate the developmental progression of cells along the temporal axis. Pseudotime analysis delineated the developmental order from left to right, with the emergence of five branches in the intermediate stage (Figure S3E, Supporting Information). Nine cell populations were identified (Figure S3F). State1 predominantly comprised CCR2^+^ macrophages, whereas State10 was predominantly populated with CCR2^‐^ macrophages. The early developmental stages were characterized by a predominance of CCR2^+^ macrophages, which persisted throughout the subsequent developmental phases, whereas CCR2^‐^ macrophages were primarily evident in the terminal stages of development. These findings suggest that CCR2^+^ cells develop earlier than CCR2^‐^ cells (Figure S3G, Supporting Information). By arranging the data in ascending order based on quasi‐timing trajectory differentiation, we identified distinct gene clusters associated with the quasi‐timing trajectories. Subsequently, we generated a heat map illustrating the dynamic patterns of gene expression changes throughout the quasi‐chronological trajectory. Genes such as Ccr2 and Cd52 demonstrated increased expression levels in the early phases of the pseudotime trajectory, signifying their pivotal roles in the development of CCR2^+^ macrophages (Figure S3H, Supporting Information). The KEGG differential genes identified between the HFD‐U and CD‐U groups of CCR2^+^ macrophages were predominantly enriched in pathways related to metabolism (Figure 3C).

These results suggest that the effect of NAFLD on metabolic pathways may be related to the role of immune cells in renal fibrosis.

### 2.4 Regulatory Role of CCR2^+^ Macrophages on Th17 Cells in NAFLD Mice Following UUO

**Figure 4:**
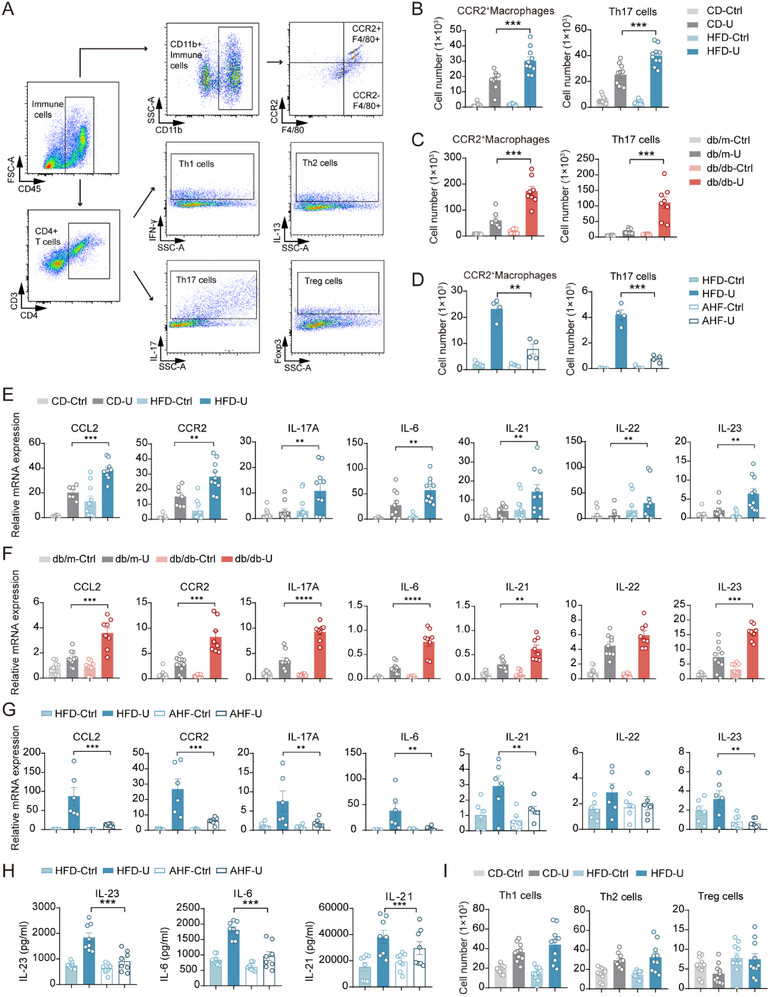
Regulatory role of CCR2^+^ macrophages on Th17 cells in mice with NAFLD following UUO. A) Identification of different macrophage and T‐cell subpopulations using various antibody combinations. Here, we analyzed immune cells using flow cytometry to identify distinct types of macrophages and CD4^+^ T cells in a mouse model of nonalcoholic fatty liver UUO. B) In both NAFLD and control mice, the number of CCR2^+^ macrophages and Th17 cells in UUO kidneys (HFD‐U, CD‐U) and control kidneys (HFD‐Ctrl, CD‐Ctrl) was quantified. C) The number of CCR2^+^ macrophages and Th17 cells in UUO kidneys (db/db‐U, db/m‐U) and control kidneys (db/db‐Ctrl, db/m‐Ctrl) of NAFLD and control mice were quantified. D) Flow cytometry analysis of Th17 and CCR2^+^ macrophages in HFD mice (HFD‐Ctrl and HFD‐U) and HFD mice treated with CCR2 inhibitors (AHF‐Ctrl and AHF‐U). E) RT‐qPCR analysis to determine the mRNA expression levels of inflammation‐related genes in kidney tissues from HFD‐ and CD‐Ctrl mice. F) RT‐qPCR analysis was performed to determine the mRNA expression levels of inflammation‐related genes in kidney tissues from db/db and db/m mice. G) RT‐qPCR analysis verifying the mRNA expression levels of inflammation‐related genes in kidney tissues from HFD and AHF mice. H) ELISA measurement of IL‐23, IL‐6, and IL‐21 protein levels in kidney tissue. I, Quantification of Th1, Th2, and Treg cells in NAFLD and control mice. B‐I: The data are presented as the means ± SEM (*n* = 4–12). Group comparisons were analyzed using a two‐tailed Student's T‐test; *p < 0.05, **p < 0.01, ***p < 0.001.

Under normal physiological conditions, macrophages are present in various tissues, including kidney macrophages, a group of long‐term resident macrophages, and those derived from circulating monocytes produced in the bone marrow.^[^ ^36^ ^]^ In the kidneys, CD4^+^T cells differentiate into proinflammatory Th1 and Th17 cells, anti‐inflammatory Th2 cells, and Tregs. ^[^ ^37^ ^]^ However, there is limited research on how renal immune cells induce kidney fibrosis in HFD‐induced NAFLD mouse models. To further explore the immunological mechanisms underlying the differences in renal fibrosis between control and NAFLD mice after UUO, we used flow cytometry to analyze the levels of macrophages and T‐cell subsets in kidney immune cells (**Figure** **4**A). The results showed a significant difference in CCR2^+^ macrophages, with a notable increase in the number of CCR2^+^ macrophages in the kidneys of the HFD‐U and db/db‐U groups (Figure 4B,C) (*p <* 0.05). Flow cytometric analysis of Th cells revealed significant changes in Treg and Th17 cell levels (*p <* 0.05) (Figure 4I), with the greatest statistical difference observed in Th17 cells (Figure 4B,C). To investigate the changes in inflammatory factors in the renal tissue, we further assessed these factors and found that, at the mRNA level, inflammatory factors associated with Th17 cell differentiation and proliferation, such as IL‐17A, IL‐6, IL‐21, IL‐22, and IL‐23, were significantly upregulated. Moreover, CCL2, an inflammatory factor associated with the recruitment of CCR2^+^ macrophages, and CCR2 receptor levels were significantly increased (*p <* 0.05) (Figure 4E,F).

Combining these findings with the single‐cell sequencing results, we statistically analyzed the number and strength of cell interactions and discovered that the CCR2^+^ subset exhibited both higher numbers and stronger interactions than the CCR2^‐^ subset. The interactions between CCR2^+^ macrophages and Th17 cells were significantly higher in the HFD‐U group than in the CD‐U group, suggesting potential communication between these two cell types (Figure 3F,G). We then used single‐cell data to analyze the receptor‐ligand relationships between CCR2^+^ macrophages and T‐cell subsets and found a significant upregulation of receptor‐ligand interactions in CCR2^+^ macrophages in the HFD‐U group (Figure 3H).

Previous studies have shown that in sepsis, CD4^+^ T cells bind to macrophages via MHC II to downregulate TLR‐induced proinflammatory signaling.^[^ ^38^ ^]^ In tumors, macrophages promote the conversion of Th1 cells to Th2 cells and influence Treg cell proliferation, migration, and function through various mechanisms.^[^ ^39^, ^40^ ^]^ In renal tissue, macrophages stimulate helper T cells to exacerbate kidney inflammation.^[^ ^41^ ^]^ These studies highlight the crucial regulatory role of macrophages in the proliferation, activation, and function of helper T cells in several diseases. Therefore, the question arises as to whether CCR2^+^ macrophages play a regulatory role in Th17 cells. To explore the regulatory role of CCR2^+^ macrophages on Th17 cells, we treated HFD mice after UUO with the CCR2 inhibitor RS504393 by oral administration for seven consecutive days and then collected kidney samples (Figure S4A, Supporting Information). Flow cytometric analysis confirmed the successful depletion of CCR2^+^ macrophages in the kidneys of HFD‐U mice (Anti‐CCR2 HFD‐U and AHF‐U) after treatment (Figure 4D). Furthermore, compared to the HFD‐U group, the AHF‐U group exhibited a significant reduction in the number of Th17 cells in the kidneys (Figure 4D), and the mRNA expression levels of fibrosis markers, such as α‐SMA, Fibronectin, and Collagen1 were significantly decreased (Figure S4B, Supporting Information). ELISA revealed a significant reduction in the expression of inflammatory factors, such as IL‐23 and IL‐6, in the kidney tissue of the AHF‐U group (Figure 4H), consistent with the changes at the mRNA level (Figure 4G).

These results validate that CCR2^+^ macrophages regulate Th17 cells by secreting inflammatory factors, such as IL‐6 and IL‐23, which promote Th17 cell proliferation and differentiation, exacerbating renal fibrosis in NAFLD mice with UUO.

### 2.5 The Linoleic Acid Metabolism Pathway Plays a Crucial role in CCR2^+^ Macrophages, with ALOX5AP Being a Key Gene that Activates this Pathway

The experiments revealed a notable increase in the populations of CCR2^+^ macrophages and Th17 cells within the kidneys following UUO in NAFLD mice compared to normal diet‐fed mice. Single‐cell sequencing revealed alterations in the metabolic profiles of the kidneys in NAFLD. We used CCR2^+^ macrophages in the kidneys of both HFD‐U and db/db‐U mice for untargeted metabolomic sequencing. Using this approach, we identified differential metabolites in two ionization modes: positive ion mode (POS) and negative ion mode (NEG). Orthogonal Least Partial Squares Discriminant Analysis (OPLS‐DA) was used to examine the distribution of samples and discern differences between the experimental groups (Figure s S5A–D, Supporting Information). Load and VIP value plots were used to identify the metabolites that contributed most significantly to the observed alterations in metabolite patterns between the comparison groups. These analyses facilitated the identification of key variables driving the principal components of the OPLS‐DA model (Figure S5G,H, Supporting Information).

**Figure 5:**
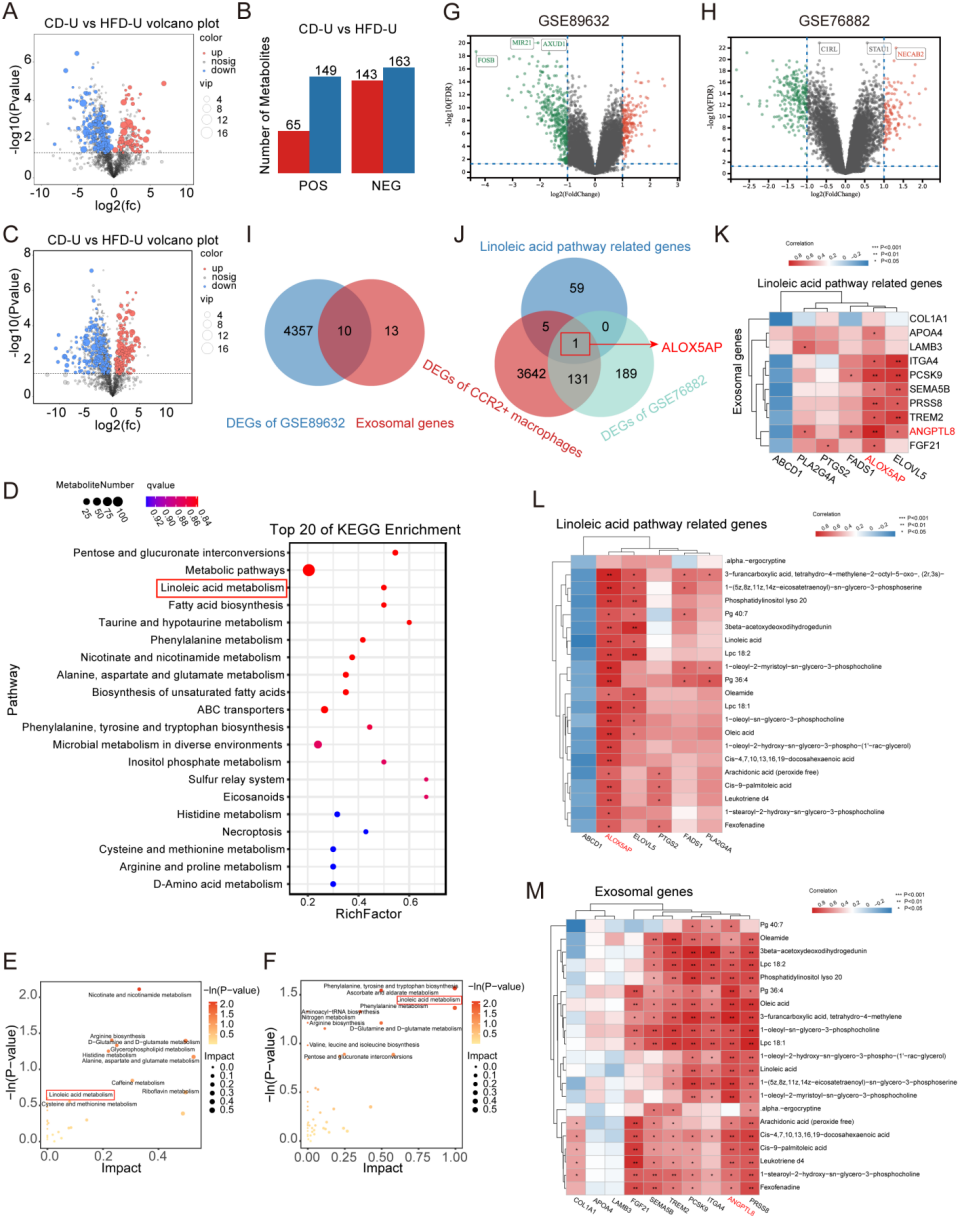
Integrated transcriptomic and metabolomic analyses of CCR2^+^ macrophages identify linoleic acid pathway genes. A) Volcanic plot of differential metabolites in POS. The abscissa is the value of the difference multiple of metabolite abundance in each comparison group after log2; the ordinate is the value of P after the T‐test after ‐log10, and the dotted line perpendicular to the Y‐axis is the threshold of the P value for differential metabolite screening. The red dots represent the upregulated differential metabolites with VIP ≥ 1 and *p <* 0.05 (FC > 1), and the blue dots represent the downregulated differential metabolites with VIP ≥ 1 and *p <* 0.05 (FC< ‐1). The larger the dot, the greater the VIP value of the metabolite. B) Quantitative statistical diagram of differential metabolites in the POS and NEG. C) Volcanic diagram of differential metabolites in NEG. D, KEGG enrichment bubble chart of the top20 in POS and NEG. The ordinate represents the pathway, and the abscissa represents the enrichment factor (the number of differential metabolites in the pathway divided by all the metabolites in the pathway). The size indicates the number; the redder the color, the smaller the Q value. E MetPA pathway analysis of differential metabolites in POS. The X‐axis represents the pathway impact, and the Y‐axis represents the pathway enrichment. Each node indicates a significantly altered metabolite cluster, with larger sizes and darker colors representing higher pathway enrichment and impact. F) MetPA pathway analysis of differential metabolites in NEG. G) Volcano plot of DEGs between nonalcoholic fatty liver disease (NAFLD) and normal liver in the dataset GSE89632. The red and green dots represent upregulated and downregulated genes, respectively. H) Volcano plot of DEGs between fibrotic and normal kidneys in the dataset GSE76882. I) Intersection of differentially expressed genes from the dataset GSE89632 and liver exocrine protein genes. J) Intersection of linoleic acid metabolism‐related genes, CCR2^+^ macrophage differential genes, and DEGs from the dataset GSE76882. K) Correlation heatmap between liver exocrine proteins and linoleic acid pathway‐related genes. L) Correlation heatmap of linoleic acid pathway‐related genes and their metabolites. M) Correlation heat map of liver exocrine protein genes and metabolites.

Following an approach integrating multivariate statistical analysis via OPLS‐DA VIP values and univariate statistical analysis using T‐test P‐values, we aimed to identify differential metabolites across various comparison groups, using a threshold of VIP ≥ 1 and T‐test *p <* 0.05 in the OPLS‐DA model, visualized through volcano diagrams. In the POS mode, 214 differential metabolites were identified, of which 65 were upregulated and 149 were downregulated (**Figure** **5**A,B). In the NEG mode, 306 differential metabolites were identified, comprising 143 upregulated and 163 downregulated metabolites (Figure 5B,C). Subsequently, we conducted a cluster analysis focusing on the top 20 differential metabolites with the highest VIP values in both ionization modes. These findings were visually represented using heatmaps (Figure S5E,F, Supporting Information).

In our study, we conducted KEGG pathway analysis of the metabolites identified in both the POS and NEG modes. We present the top 20 pathways with the smallest Q values using bubble plots. Metabolic pathways affected by the disturbances induced by HFD consumption in both modes included pentose and gluconate interconversion, linoleic acid metabolism, and fatty acid biosynthesis (Figure 5D). Furthermore, we performed topological analysis using metrics such as degree centrality and relative betweenness centrality to ascertain the significance of differential metabolite‐enriched signaling pathways within the network diagram. This analysis was complemented by a hypergeometric or Fisher's exact test to determine the significance of the pathways, helping to identify pathways with critical positional information (Figure 5E,F). Consistent with the KEGG signaling pathway analysis, the linoleic acid metabolism pathway was ranked among the top three pathways in terms of IMPACT values in the NEG model. This pathway was enriched in six differential metabolites, with linoleic and alpha‐linolenic acids exhibiting the most significant differences. This further suggests that in mice with NAFLD and concurrent kidney fibrosis, linoleic acid metabolism plays an important role in regulating the function of CCR2^+^ macrophages.

We further explored the pathway genes and downstream metabolites using single‐cell transcriptomics and untargeted metabolomic sequencing (Figure S6A, Supporting Information). First, we conducted a transcriptomic analysis to identify key pathway genes. Differential analysis was performed for the normal and NAFLD groups in the GSE89632 cohort (Figure 5G). The intersection of the excretory protein genes in the liver and the differentially expressed genes led to the selection of the top 10 genes with the highest positive fold changes as excretory protein genes that promote NAFLD in the liver (Figure 5I). By intersecting linoleic acid metabolism pathway‐related genes (Table S1, Supporting Information) with differentially expressed genes in CCR2^+^ macrophages, we identified six linoleic acid metabolism pathway genes expressed in renal macrophages. Among them, PTGS2 and ALOX5AP showed a fold change greater than 1 (*p <* 0.05), indicating the high expression of PTGS2 and ALOX5AP in CCR2^+^ macrophages. To further validate the accuracy of these two genes, we selected the GSE76882 cohort as a validation cohort. After performing differential analysis between the normal and renal fibrosis groups in this cohort (Figure 5H), we intersected these results with the six linoleic acid metabolism pathway genes. We found that ALOX5AP exhibited the most significant differential expression and was associated with the promotion of renal fibrosis, with a fold change greater than 1 and *p <* 0.05, suggesting that ALOX5AP is a key pathway gene (Figure 5J). Next, we performed metabolomic analysis to identify the key downstream metabolites. First, differential analysis was performed on the tissue metabolomics data, selecting the 20 metabolites with the most significant differences in expression that were highly expressed in the HFD group (Figure S5E,F, Supporting Information). We then conducted a correlation analysis between the 10 excretory protein genes in the liver and six linoleic acid metabolism‐related genes and visualized the results using a heatmap. The analysis revealed the strongest correlation between ANGPTL8 and ALOX5AP, suggesting that ANGPTL8 may be an upstream gene that triggers the linoleic acid metabolism pathway (Figure 5K). By correlating the six linoleic acid metabolism‐related genes measured in tissues with 21 metabolites associated with the linoleic acid metabolism pathway and visualizing the results using a heatmap, we identified the downstream metabolites with the strongest correlations (Figure 5L). Finally, we verified the downstream pathway metabolites by performing a correlation analysis between the 10 liver excretory protein genes and 21 metabolites, and obtained similarly strong correlations for the downstream metabolites (Figure 5M). A dendrogram illustrating the metabolic differences in the linoleic acid metabolic pathway between the NAFLD and control groups was generated (Figure S6B, Supporting Information).

Based on single‐cell transcriptomics and untargeted metabolomic sequencing analysis of mice with NAFLD and renal fibrosis, we predicted that the liver excretory protein ANGPTL8 acts as a bridge in the progression of kidney fibrosis exacerbated by NAFLD. ANGPTL8 activates ALOX5AP in CCR2^+^ macrophages, inducing the expression of downstream metabolites of the ALOX5 pathway.

### 2.6 Inflammatory Response of CCR2^+^PIRB^+^ Macrophages and Enhanced Linoleic Acid Metabolism

Previous studies have shown that ANGPTL8 is highly expressed in steatotic liver cells and binds to PIRB receptors on macrophages, promoting liver injury and fibrosis.^[^ ^23^, ^42^ ^]^ However, the interaction between ANGPTL8 and PIRB, as well as its relationship with ALOX5AP and linoleic acid metabolism in mice with NAFLD and concurrent renal fibrosis, has not been thoroughly investigated.

**Figure 6:**
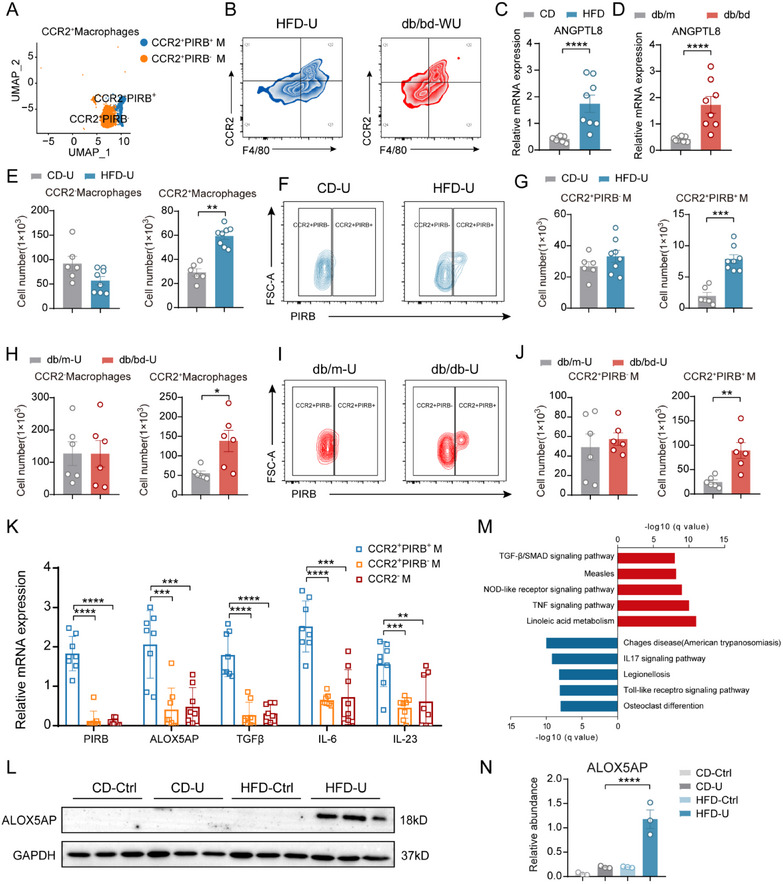
Elevated CCR2^+^PIRB^+^ macrophages in renal tissues induced by NAFLD. A) UMAP dimensionality reduction plot of CCR2^+^ macrophages in the NAFLD group, showing two subpopulations with distinct markers: CCR2^+^PIRB^+^ and CCR2^+^PIRB^‐^ macrophages. B) Flow cytometry identification of CCR2^+^PIRB^+^ and CCR2^+^PIRB^‐^ macrophages in the renal tissues of NAFLD mice after UUO surgery. C) mRNA levels of ANGPTL8 in the livers of HFD and CD mice. D) mRNA levels of ANGPTL8 in the livers of db/db and db/m mice. E) Quantification of CCR2⁺ and CCR2^−^ macrophages in fibrotic kidneys of HFD and CD mice after renal‐fibrosis modeling. F) Representative flow cytometry plots of CCR2⁺PIRB⁺ and CCR2⁺PIRB^−^ macrophages in fibrotic kidneys from HFD and CD mice. G) Quantification of data from panel F. H) Quantification of CCR2^+^ and CCR2^‐^ macrophages in the UUO kidneys of db/db and db/m mice. I) Representative flow cytometry plots of CCR2^+^PIRB^+^ and CCR2^+^PIRB^‐^ macrophages in UUO kidneys of db/db and db/m mice. J) Quantification of data from panel I. K) mRNA levels of linoleic acid metabolism‐related genes and inflammatory factors in different cell populations from panel B. L) Western blot analysis of ALOX5AP protein expression in UUO‐operated kidneys and healthy kidneys from HFD and CD mice. M) KEGG enrichment analysis of differentially expressed genes in CCR2^+^PIRB^+^ macrophages from panel A. N) Quantification of ALOX5AP protein levels from panel L (*n* = 3). C, D, E, G, H, J, K, N) The data are presented as the means ± SEM (*n* = 6–8). Group comparisons were analyzed using a two‐tailed Student's T‐test and ANOVA with Turkey's multiple comparisons test; * *p <* 0.05, ** *p <* 0.01, *** *p <* 0.001, **** *p <* 0.0001.

Single‐cell sequencing results indicated that CCR2^+^ macrophages can be divided into two groups: CCR2^+^PIRB^+^ and CCR2^+^PIRB^‐^ macrophages (**Figure** **6**A). In the NAFLD model induced by HFD feeding and in db/db mice with renal fibrosis, we observed that CCR2^+^ macrophages were separated into two groups (Figure 6B). To further validate this, we used flow cytometry to identify both CCR2^+^ and CCR2^‐^ macrophages and further classified CCR2^+^ macrophages into CCR2^+^PIRB^+^ and CCR2^+^PIRB^‐^ macrophages (Figure 6E,F). In the kidneys of normal diet‐fed mice with fibrosis, the number of CCR2^+^ macrophages increased, predominantly driven by an increase in the number of CCR2^+^PIRB^‐^ macrophages. However, in the kidneys of HFD‐induced NAFLD mice with fibrosis, the number of CCR2^+^PIRB^+^ macrophages was significantly higher than that in the fibrotic kidneys of normal diet‐fed mice (Figure 6G). This phenomenon was also observed in another NAFLD model (Figure 6H–J), suggesting that CCR2^+^PIRB^+^ macrophages may be regulated by NAFLD‐specific conditions in mice. We further discovered that in both HFD‐fed and db/db gene‐induced NAFLD mice, the mRNA levels of ANGPTL8 in the livers of mice were significantly elevated, consistent with previous findings (Figure 6C,D). Upon sorting the macrophages from the above subgroups and performing mRNA level analysis, we found that CCR2^+^PIRB^+^ macrophages exhibited a stronger linoleic acid metabolism ability (with increased ALOX5AP expression), enhanced capacity to promote Th17 cell proliferation and differentiation (with increased IL‐6 and IL‐23 expression), and greater fibrosis‐promoting ability (with elevated TGF‐β expression) (Figure 6K). Consistent with the mRNA levels, the protein levels of ALOX5AP were significantly elevated in the kidney tissues of NAFLD mice with renal fibrosis (Figure 6L,N). Enrichment analysis of the differentially expressed genes in CCR2^+^PIRB^+^ macrophages further supported our findings (Figure 6M). This suggests that CCR2^+^PIRB^+^ macrophages are the key immune cells that exacerbate renal fibrosis in patients with NAFLD.

### 2.7 ANGPTL8/PIRB Regulates ALOX5AP Expression in Macrophages, Inducing the Upregulation of Inflammatory Factors

To validate the protein levels of ANGPTL8 in NAFLD mice, we performed ELISA and found that ANGPTL8 levels were significantly higher only in the liver and white adipose tissue homogenates of mice (Figure S7A,B, Supporting Information). To exclude the potential influence of proteins secreted by organs other than the liver in NAFLD mice, we cultured BMDM cells with homogenates from various tissues and assessed intracellular ALOX5AP levels using flow cytometry. The results revealed that only liver homogenates markedly increased the proportion of ALOX5AP^+^ macrophages (Figure S7C,D, Supporting Information). This experiment ruled out the effects of other organs on CCR2^+^PIRB^+^ macrophages in the kidneys, confirming that only secretory proteins in the liver play a crucial role in activating CCR2^+^PIRB^+^ macrophages in the kidneys.

**Figure 7:**
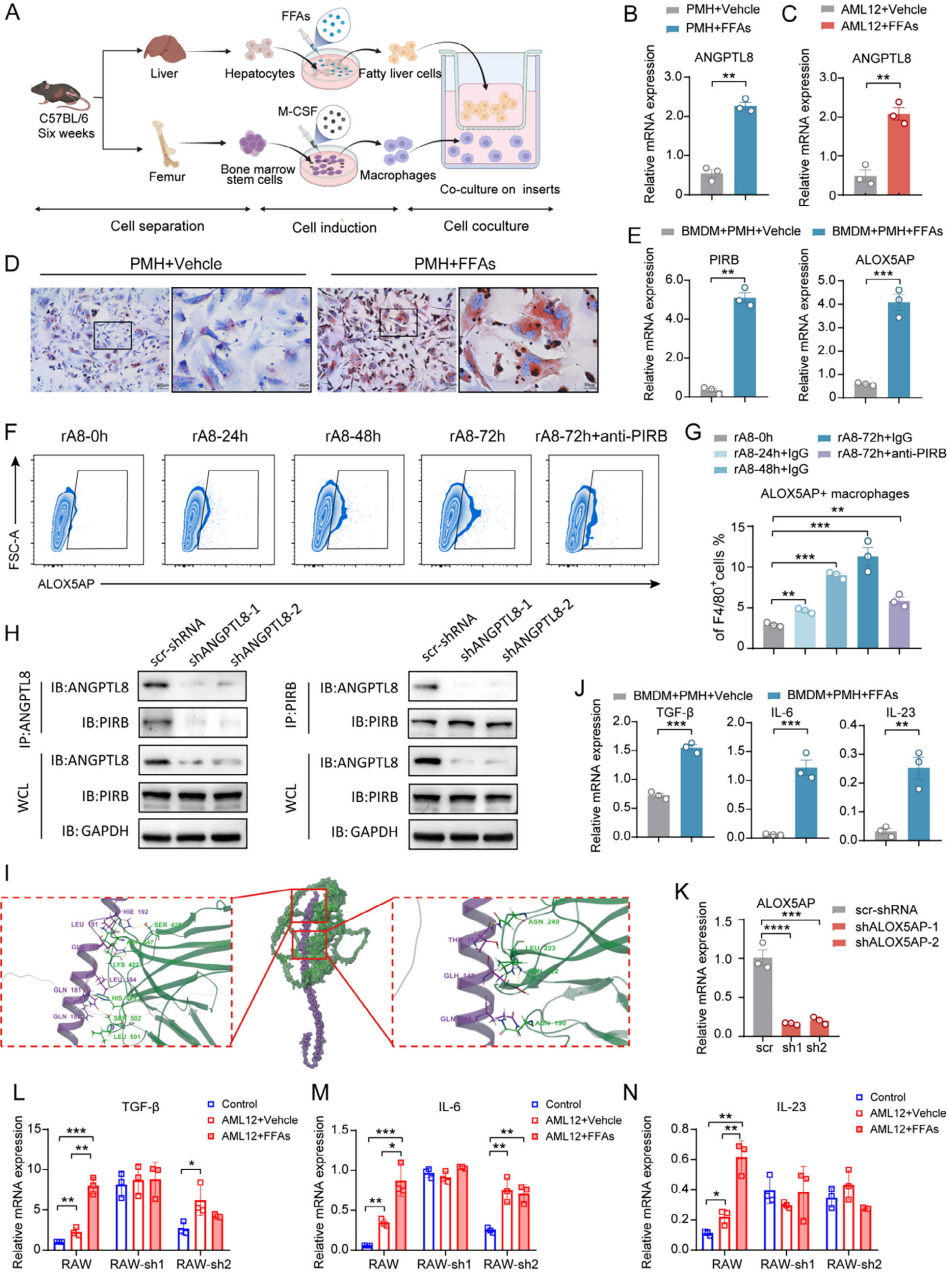
ANGPTL8/PIRB regulates ALOX5AP expression in macrophages. A) Schematic diagram of primary liver cell fat accumulation induction, in vitro induction of bone marrow‐derived macrophages (BMDMs), and coculture. B) mRNA expression levels of ANGPTL8 in PMH after treatment with Vehicle or FFAs (*n* = 3). C) mRNA expression levels of ANGPTL8 in mouse liver cells (AML12) after treatment with vehicle or FFAs (*n* = 3). D) Representative images of Oil Red O staining showing lipid droplets in fat‐accumulated PMH cells in vitro. Left: 100x magnification; Right: 400x magnification of the black‐bordered area. E) ALOX5AP, and PIRB mRNA levels in BMDMs were measured after a 48‐hour noncontact coculture with hepatocytes. F) ALOX5AP expression in BMDMs after treatment with recombinant ANGPTL8 (rA8) and anti‐PIRB neutralizing antibody at different time points, as analyzed by flow cytometry. G, Quantification of ALOX5AP expression (F). H) Immunoprecipitation and Western blot analysis revealed the binding of ANGPTL8 to PIRB on BMDM cells. I) Molecular docking results of ANGPTL8 (purple) with PIRB (green), with the potential binding regions highlighted in the red box. J) mRNA levels of inflammatory factors in BMDMs were measured after a 48‐hour noncontact coculture with hepatocytes (*n* = 3). K) Determination of ALOX5AP mRNA levels in ALOX5AP‐knockdown RAW264.7 cells using qRT‐PCR. L─N) mRNA levels of various genes and inflammatory factors in RAW264.7 cells from the ALOX5AP knockdown group and negative control group. B,C,E,G,J,K,L‐N) The data are presented as the means ± SEM (*n* = 3). Group comparisons were analyzed using a two‐tailed Student's T‐test and ANOVA with Turkey's multiple comparisons test; * *p <* 0.05, ** *p <* 0.01, *** *p <* 0.001, **** *p <* 0.0001. PMH, primary mouse hepatocytes; BMDMs: Bone marrow‐derived macrophages; rA8, recombinant ANGPTL8 protein.

We extracted primary mouse hepatocytes (PMH) and established an in vitro NAFLD model using conditioned medium. We isolated mouse bone marrow stromal cells and induced the generation of BMDMs with M‐CSF stimulation (**Figure** **7**A). After treating liver cells with free fatty acids (FFAs) for 24 h, Oil Red O staining showed a significant increase in lipid droplet abundance (Figure 7D), and ANGPTL8 mRNA levels were notably elevated (Figure 7B). To investigate the direct effect of ANGPTL8 on BMDMs, we added recombinant ANGPTL8 (rANGPTL8) protein to the BMDMs culture medium at different time points and monitored changes in ALOX5AP expression (Figure 7F). ALOX5AP levels increased progressively with prolonged exposure to rANGPTL8 and peaked at 72 h. Upon the addition of anti‐PIRB, a neutralizing antibody against the ANGPTL8 receptor, ALOX5AP expression decreased significantly (Figure 7G), indicating that ANGPTL8 activates ALOX5AP expression by binding to the PIRB receptor of the BMDMs. We subsequently performed a co‐immunoprecipitation (Co‐IP) assay and discovered that the PIRB protein in BMDM cells could bind to exogenous ANGPTL8 protein (his‐tag) (Figure S7E, Supporting Information). Furthermore, we induced Steatotic AML12 cell line and knocked down ANGPTL8 expression (Figure S7G,H, Supporting Information). After concentrating the culture medium through ultrafiltration and co‐culturing it with BMDM cells, we observed a significant reduction in the binding of these two proteins upon ANGPTL8 gene knockdown (Figure 7H). Moreover, the expression of ALOX5AP in BMDM cells was also notably decreased (Figure S7F, Supporting Information). To validate their binding domains, we conducted molecular docking predictions between PIRB and ANGPTL8. Our findings revealed that the HIE192 residue of ANGPTL8 forms a hydrogen bond with ASP447 and SER425 of PIRB, while LEU191 of ANGPTL8 interacts with ASP447 of PIRB, and GLN188 of ANGPTL8 forms a hydrogen bond with LYS422 of PIRB. These interactions indicate that ANGPTL8 and PIRB can bind together in a highly stable manner (PIPER pose energy <–1000 kcal mol^−1^) (Figure 7I).

This indicates that ANGPTL8 can activate ALOX5AP expression by binding to the PIRB receptor on BMDM cells. But how does this interaction influence the kidney in mice with NAFLD complicated by renal fibrosis? To simulate the liver‐kidney axis, we performed a noncontact coculture experiment between PMH and BMDMs and measured the mRNA levels of linoleic acid metabolism‐related ALOX5AP, ANGPTL8 receptor PIRB, and inflammatory factors TGF‐β, IL‐6, and IL‐23 associated with fibrosis, Th17 cell proliferation, and differentiation. The expression levels of these genes were significantly higher in the NAFLD model coculture group than in the control coculture group (Figure 7E,J). We replicated the coculture experiment using RAW 264.7 and AML12 cells (Figure 7C) and performed ALOX5AP knockdown in RAW 264.7 cells (Figure 7K). Despite the NAFLD model coculture, the mRNA levels of TGF‐β, IL‐6, and IL‐23 remained unchanged ((Figure 7L–N), highlighting the crucial regulatory role of linoleic acid metabolism‐related kinase ALOX5AP in modulating these inflammatory factors.

### 2.8 CCR2^+^PIRB^−^ Macrophages and CCR2^+^PIRB⁺ Macrophages Promote Renal Fibrosis by Regulating Fibroblast Function Through Secretion of Distinct Cytokines

**Figure 8:**
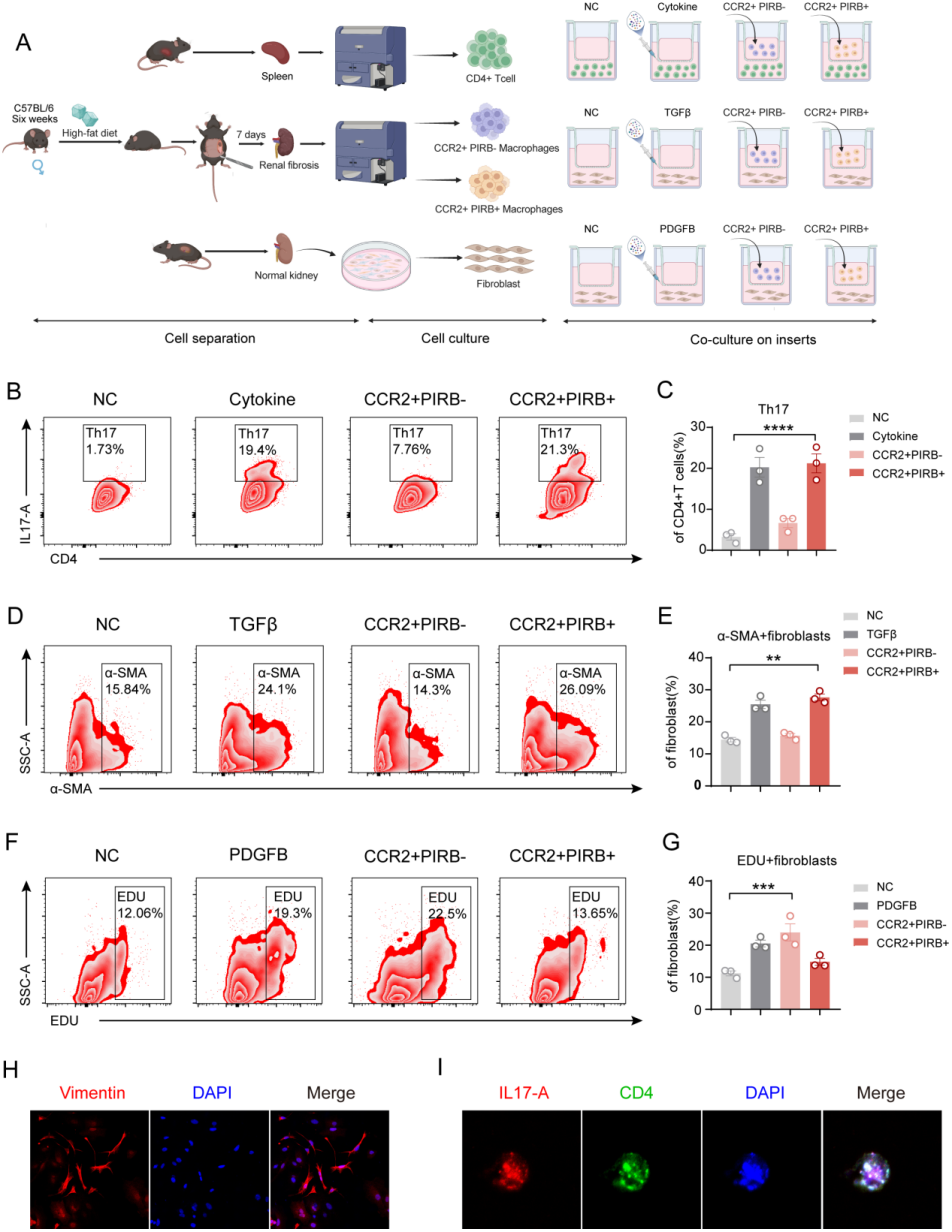
CCR2^+^PIRB^‐^ and CCR2^+^PIRB^+^ macrophages regulate fibroblast proliferation and fibrogenesis. A) Schematic representation of the in vitro coculture system: primary renal CCR2⁺PIRB^−^ macrophages versus CCR2⁺PIRB^−^ macrophages with primary fibroblasts. B) IL‐17 expression in primary CD4⁺ T cells cultured under normal control (NC), Th17‐polarizing cytokines, or co‐cultured with CCR2⁺PIRB⁺/CCR2⁺PIRB^−^ macrophages for 3 days. Flow cytometry analysis. C) Quantification of IL‐17⁺CD4⁺ T cells from panel B. D) α‐SMA expression in primary fibroblasts treated with NC, TGF‐β (fibrotic induction), or cocultured with CCR2⁺PIRB⁺/CCR2⁺PIRB^−^ macrophages for 7 days. Flow cytometry analysis. E) Quantitative analysis of α‐SMA⁺ fibroblasts from panel D. F) EdU incorporation assay in primary fibroblasts exposed to: NC, PDGF‐BB (proliferative induction), or cocultured with CCR2⁺PIRB⁺/CCR2⁺PIRB^−^ macrophages for 3 days. G) Quantification of EdU⁺ fibroblasts from panel F. H) Immunofluorescence characterization of primary fibroblasts isolated from murine kidney. I) IL‐17 immunofluorescence in CD4⁺ T cells after coculturing with CCR2⁺PIRB^−^. C,E,G) The data are presented as the means ± SEM (*n* = 3). Group comparisons were analyzed using ANOVA with Turkey's multiple comparisons test; **p < 0.01, ***p < 0.001, ****p < 0.0001.

To explore the regulatory roles of CCR2^+^PIRB^‐^ and CCR2^+^PIRB^+^ macrophages in renal fibrosis and T‐cell inflammation, we established coculture systems with primary renal macrophages and primary fibroblasts, as well as primary renal macrophages and primary CD4^+^ T cells (**Figure** **8**A). First, we isolated primary CD4^+^ T cells from mouse spleens using flow cytometry sorting and obtained primary renal fibroblasts through selective culture. The identity of these cells was confirmed by immunofluorescence (Figure 8H,I). Next, we divided the primary CD4^+^ T cells into four treatment groups: the negative control group using standard culture medium, the positive control group with Th17 polarization factors, and experimental groups using CCR2^+^PIRB^‐^ and CCR2^+^PIRB^+^ macrophages (Figure 8B). After three days of coculture, we found that the expression of IL‐17 was significantly elevated in CD4^+^ T cells co‐cultured with CCR2^+^PIRB^+^ macrophages, whereas the CCR2^+^PIRB^‐^ macrophage group showed minimal changes (Figure 8C), further confirming that CCR2^+^PIRB^+^ macrophages promote Th17 cell differentiation. We assessed the effect of these two types of macrophages on fibroblasts. Based on previous experiments showing that CCR2^+^PIRB^+^ macrophages secrete fibrosis‐inducing factors such as TGFβ, we used TGFβ as a positive control to observe the degree of fibrosis in fibroblasts after coculturing with these two types of macrophages (Figure 8D). We found that the expression of α‐SMA, a marker of fibrosis, was significantly increased in fibroblasts co‐cultured with CCR2^+^PIRB^+^ macrophages, whereas no significant changes were observed in the CCR2^+^PIRB^‐^ macrophage group (Figure 8E), supporting the conclusion that CCR2^+^PIRB^+^ macrophages promote fibrosis in fibroblasts. In addition, in coculture experiments involving NAFLD PMH cells, BMDM, and fibroblasts, we found that the coculture of PMH and BMDM cells significantly enhanced fibroblast fibrosis, a process that could be blocked by ANGPTL8 neutralizing antibodies. This supports the hypothesis that CCR2^+^PIRB^+^ macrophages promote fibroblast fibrosis by transmitting ANGPTL8 signals derived from steatotic hepatocytes (Figure S8A, Supporting Information). What role do CCR2^+^PIRB^‐^ macrophages play in this process?

We extracted differential genes from CCR2^+^PIRB^‐^ macrophages using single‐cell sequencing data and performed enrichment analysis. The differentially expressed genes were significantly enriched in the cell proliferation‐related signaling pathways (Figure S8B, Supporting Information). Based on a literature review, we identified three excretory genes related to cell proliferation: IGF1, PDGFB, and PDGFA. RT‐qPCR revealed that only PDGFB showed the greatest differential expression in CCR2^+^PIRB^‐^ macrophages, which is a gene highly associated with cell proliferation (Figure S8C, Supporting Information). Next, we co‐cultured both macrophage subsets with fibroblasts and added PDGF‐B to the culture medium of the positive control. After three days of coculture, an EdU proliferation assay was performed (Figure 8F, Supporting Information). We found that the fibroblast population with EdU‐positive cells was significantly increased in the CCR2^+^PIRB^‐^ macrophage coculture group, whereas no significant changes were observed in the CCR2^+^PIRB^+^ macrophage group (Figure 8G). This indicates that CCR2^+^PIRB^‐^ macrophages promote renal fibrosis by enhancing fibroblast proliferation through PDGFB secretion.

CCR2^+^PIRB^‐^ and CCR2^+^PIRB^+^ macrophages play distinct roles in the renal immune microenvironment. CCR2^+^PIRB^‐^ macrophages promoted fibroblast proliferation, whereas CCR2^+^PIRB^+^ macrophages promoted Th17 cell polarization and fibroblast fibrosis. Taken together, these two macrophage subsets regulate fibroblast function and contribute to renal fibrosis.

### 2.9 Knockout of ALOX5AP in Mouse Monocyte‐Macrophages Reduces Th17 Cell Generation and Alleviates Kidney Fibrosis

**Figure 9:**
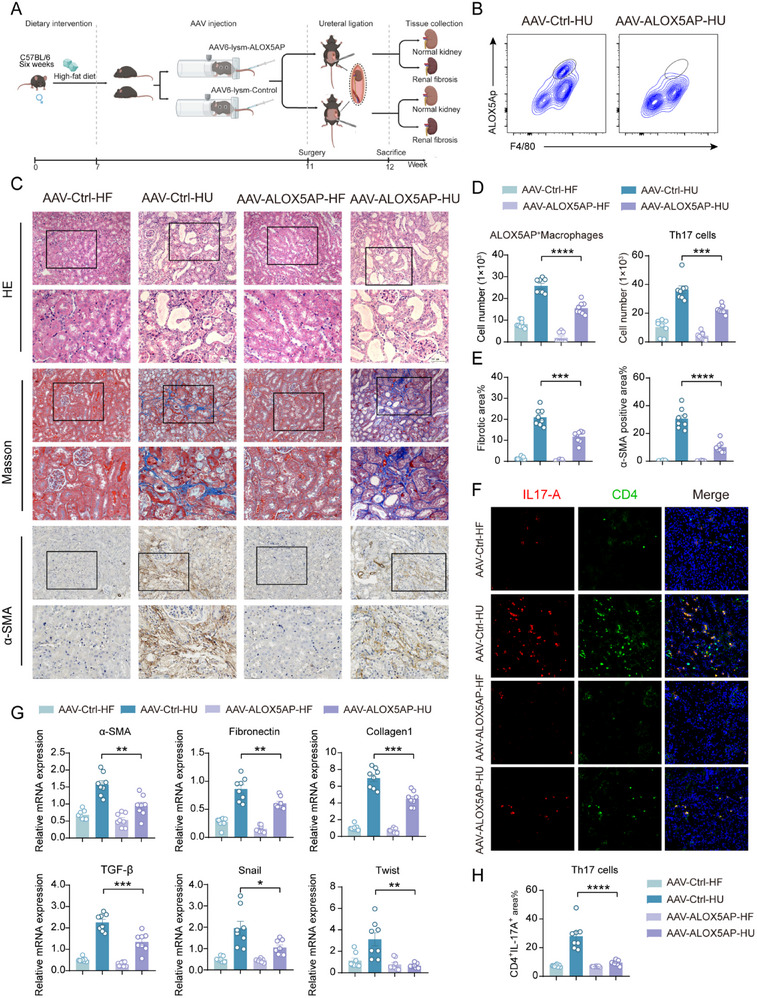
Macrophage‐specific ALOX5AP knockout via AAV delivery demonstrates therapeutic effects on renal fibrosis A) Experimental timeline for establishing NAFLD and renal‐fibrosis models with HFD mice and AAV treatment: Mice were fed a high‐fat or normal diet for 7 weeks, followed by tail vein injection of AAV. At week 11, UUO surgery was performed, and the liver and kidneys were harvested for evaluation 7 days post‐surgery. B) Representative flow cytometry plots showing ALOX5AP⁺ macrophages in the kidneys of mice subjected to UUO. C) HFD mice were divided into AAV‐lysm‐ALOX5AP and AAV‐lysm‐control groups. Seven days after UUO surgery, the right kidneys were left unlighted (AAV‐ALOX5AP‐HF and AAV‐Ctrl‐HF), and the left kidneys were ligated (AAV‐ALOX5AP‐HU, AAV‐Ctrl‐HU). Hematoxylin and eosin (HE) staining was used to evaluate kidney tissue morphology, Masson's trichrome staining was used to assess collagen deposition (blue), and immunohistochemistry was used to visualize α‐SMA expression (brown) (*n* = 10; scale bars: 100 µm, 50 µm). D) Quantitative analysis of ALOX5AP^+^ macrophages and Th17 cells. E) Quantitative data for Masson's trichrome and α‐SMA immunohistochemistry from Panel C. F) Representative images of immunofluorescence staining for IL‐17A (red) and CD4 (green) in kidney sections from the AAV‐lysm‐ALOX5AP‐treated and AAV‐lysm‐control groups. Images are shown at 200× magnification. G, RT‐qPCR analysis of mRNA expression levels of fibrosis‐related genes in kidney tissue from the AAV‐lysm‐ALOX5AP and AAV‐lysm‐control groups. H) Quantification of data from panel F. D,E,G,H) The data are presented as the means ± SEM (*n* = 8). Group comparisons were analyzed using a two‐tailed Student's T‐test; *p < 0.05, ** *p <* 0.01, *** *p <* 0.001, **** *p <* 0.0001.

We developed an AAV gene knockout mouse model to investigate the role of ALOX5AP in macrophages. Specifically, we injected AAV‐LYSM‐ALOX5AP and AAV‐LYSM‐Control viruses into the tail veins of high‐fat diet‐fed mice to create experimental and control groups with targeted knockout of ALOX5AP in monocyte macrophages in vivo. After 28 days of viral treatment, UUO surgery was performed on the mice, which were sacrificed seven days later to collect their kidneys (**Figure** **9**A). We assessed the expression of ALOX5AP in mouse kidneys using flow cytometry. The results showed that fter AAV treatment, ALOX5AP^+^ macrophages were almost completely absent from the kidneys (Figure 9B,D). Morphological analysis revealed that ureteral damage was significantly reduced in the AAV‐LYSM‐ALOX5AP group, with notable decreases in Masson's trichrome staining and α‐SMA immunohistochemical positivity (Figure 9C,E). These findings indicate that ALOX5AP knockout in renal macrophages has a therapeutic effect on kidney fibrosis. In addition, at the mRNA level, genes associated with fibrosis, such as Fibronectin, Collagen1, and α‐SMA, were significantly upregulated (Figure 9G). As previously established, ALOX5AP is a key enzyme in the linoleic acid metabolism pathway that stimulates Th17 cell polarization, contributing to fibrosis. Flow cytometry (Figure 9D) and immunofluorescence (Figure 9F,H) were performed to measure the Th17 cell numbers in the kidneys of treated mice. We found that following AAV treatment, the number of Th17 cells in the kidneys was significantly reduced, consistent with our previous coculture experiments in RAW264.7 cells in which ALOX5AP was knocked down. Subsequently, RT‐qPCR was used to measure the expression of transcription factors and inflammatory cytokines related to Th17 cell polarization in kidneys. We observed a significant decrease in the expression of the transcription factors ROR‐γt, STAT3, and NF‐κB, as well as the inflammatory cytokines IL‐6, IL‐23, and IL‐17A (Figure S9A,B, Supporting Information). These results confirm the regulatory role of ALOX5AP^+^ macrophages in Th17 cell polarization and establish ALOX5AP as a critical target for treating NAFLD with concurrent kidney fibrosis.

### 2.10 Validation of the ANGPTL8/LILRB2/ALOX5AP Axis in Renal Tissues of Patients with NAFLD and Concurrent Renal Fibrosis

**Figure 10:**
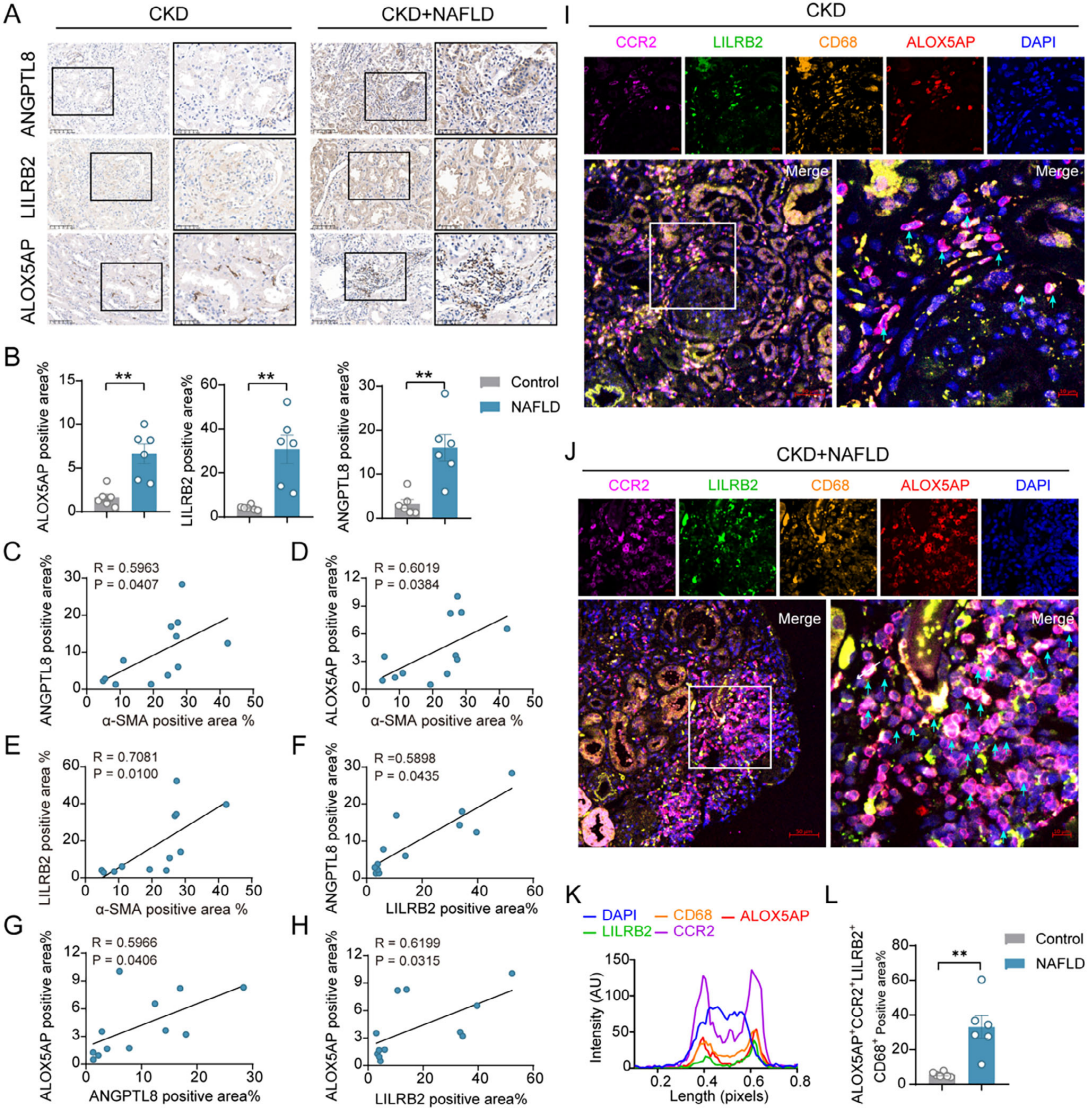
High expression of ALOX5AP in CCR2^+^LILRB2^+^ macrophages in the renal tissues of patients with NAFLD and concurrent renal fibrosis. A) Representative images showing immunohistochemical staining (brown) for α‐SMA, ANGPTL8, LILRB2, and ALOX5AP in renal tissues from patients with CKD (CKD) and CKD patients with NAFLD (CKD + NAFLD). In both groups, the left image scale bar is 100 µm, and the right image is an enlarged view of the black‐bordered area from the left image, scale bar 50 µm. B) Quantification of immunohistochemical staining for ANGPTL8, LILRB2, and ALOX5AP. C─H) Correlation analysis of immunohistochemical staining for α‐SMA, ANGPTL8, LILRB2, and ALOX5AP. I, Representative renal tissue sections from patients with CKD showing immunostaining for CCR2, LILRB2, CD68, and ALOX5AP. The lower left image is at 200× magnification, with a scale bar of 50 µm. The lower right image is at 630× magnification of the white‐bordered area, with the top image showing monochrome channels; the scale bar is 10 µm. J) Representative renal tissue sections from patients with CKD + NAFLD, showing immunostaining for CCR2, LILRB2, CD68, and ALOX5AP. The lower left image is at 200x magnification with a scale bar of 50 µm. The lower right image is at 630x magnification of the white‐bordered area, with the top image showing monochrome channels; the scale bar is 10 µm. K) Enlarged view of the white‐bordered area in panel (J) showing the intensity distribution and colocalization along the white arrow direction. L) Quantification of CCR2^+^, LILRB2^+^, CD68^+^, and ALOX5AP^+^ cells and the percentage of positive areas in tissue sections from both patient groups B,L) The data are presented as the means ± SEM (n = 6). Group comparisons were analyzed using a two‐tailed Student's *t*‐test; **p < 0.01. C─H) Correlation analyses were conducted using Pearson's correlation coefficient.

We selected a group of patients with CKD and biopsy‐confirmed renal fibrosis and categorized them into two groups: those with renal fibrosis alone (CKD) and those with renal fibrosis combined with NAFLD (CKD + NAFLD). Renal biopsy specimens were subjected to immunohistochemical analysis. The results revealed that the protein expression levels of the fibrosis marker α‐SMA were significantly higher in the CKD + NAFLD group than in the CKD group (Figure 10E,G). As a direct homolog of the mouse paired immunoglobulin‐like receptor B (PIRB), the human leukocyte immunoglobulin‐like receptor B2 (LILRB2) also plays a role in liver‐kidney axis function.^[^ ^43^ ^]^ We found that the protein expression levels of ANGPTL8, LILRB2, and ALOX5AP, which are related to the liver‐kidney axis, were significantly higher in the CKD + NAFLD group than in the CKD group (**Figure** **10**A,B). Furthermore, there was a positive correlation between ANGPTL8, LILRB2, ALOX5AP, and α‐SMA expression (Figure 10C–H). We performed multiplex immunofluorescence staining on tissues from both the CKD and CKD+NAFLD groups to characterize the immune phenotype and quantify the immune cell composition. The results showed that LILRB2 and ALOX5AP co‐localized in macrophages (Figure S10A,B,D, Supporting Information), and the number of macrophages was significantly higher in the CKD + NAFLD group (Figure S10C, Supporting Information). To further confirm whether these macrophages had a CCR2^+^ background, we performed colocalization detection of CCR2, LILRB2, and ALOX5AP (Figure 10I–K) and confirmed the presence of highly infiltrated CCR2^+^LILRB2^+^ macrophages in CKD+NAFLD kidneys with elevated ALOX5AP levels (Figure 10L). This provides spatial evidence for the ANGPTL8‐regulated CCR2^+^LILRB2^+^ macrophage linoleic acid metabolism axis and confirms the existence of the ANGPTL8/PIRB/ALOX5AP liver‐kidney axis.

## 3 Discussion

NAFLD is a prevalent chronic metabolic disorder that is increasingly recognized as a major global public health challenge. Clinical studies have consistently shown that patients with NAFLD have a significantly elevated risk of renal dysfunction and CKD, with renal fibrosis being a key pathological change in the final stages.^[^ ^44^, ^45^ ^]^ However, the exact mechanisms underlying accelerated renal fibrosis in NAFLD remain unclear. Our findings suggest that the ANGPTL8/PIRB/ALOX5AP axis plays a crucial role in liver‐kidney interactions in NAFLD. Elevated ANGPTL8 levels released by fatty liver cells enter the bloodstream and reach the kidneys, where they interact with CCR2^+^PIRB^+^ macrophages, driving macrophage‐mediated inflammation and fibrosis by reprogramming linoleic acid metabolism.

ANGPTL8 is a recently discovered member of the ANGPTL family. Numerous studies have reported increased hepatic ANGPTL8 expression in NAFLD mouse models.^[^ ^22^, ^46^ ^]^ As a secreted protein, it is highly expressed in human liver tissue and secreted into the bloodstream. Recent studies have also revealed the involvement of ANGPTL8 in inflammatory diseases, which is consistent with our single‐cell transcriptomic sequencing and untargeted metabolomic analyses.^[^ ^20^ ^]^ Therefore, we hypothesized that ANGPTL8 plays a critical role in the exacerbation of kidney fibrosis in patients with NAFLD. This finding marks a breakthrough in overcoming the limitations of previous studies that primarily linked serum ANGPTL8 levels to liver macrophage PIRB‐mediated liver fibrosis.^[^ ^23^ ^]^ We demonstrated that ANGPTL8, an extrahepatic effector, drives kidney fibrosis via macrophage PIRB signaling, a mechanism that is distinct from its autocrine/paracrine action in the liver.

Single‐cell analysis revealed novel dimensions of functional heterogeneity in profibrotic renal CCR2^+^ macrophages under NAFLD stress. Classical theory suggests that renal CCR2^+^ macrophages originate from monocytes and persistently co‐express F4/80 and CCR2 after infiltrating tissues.^[^ ^28^ ^]^ We identified and differentiated two functional subsets based on the PIRB expression. In the fibrotic kidneys of the control group (normal diet), CCR2^+^PIRB^‐^ macrophages dominated, whereas in the NAFLD group, there was a significant shift toward CCR2^+^PIRB^+^ macrophages. This reversal of macrophage subsets in NAFLD highlights the role of CCR2^+^PIRB^+^ macrophages as key responders to hepatic signals such as ANGPTL8. This discovery challenges the traditional view of homogeneous function among CCR2^+^ macrophages in renal fibrosis and echoes the emerging evidence of macrophage heterogeneity in metabolic organ interactions (e.g., similar transitions reported in diabetic cardiomyopathy).

Fibrosis is an unresolved inflammatory response; however, studies on the systemic immune‐metabolic interactions that drive NAFLD‐induced renal fibrosis remain insufficient. By integrating single‐cell and metabolomic data, we established, for the first time, that CCR2^+^PIRB^+^ macrophages play a central role in coordinating linoleic acid metabolism regulated by the 5‐LOX/FLAP complex. Specifically, ANGPTL8‐activated macrophages reprogram arachidonic acid metabolism (an upstream pathway of proinflammatory eicosanoid biosynthesis) to adopt an inflammatory phenotype. Specific lipid metabolic reprogramming in macrophages and its association with the excessive production of IL‐6/IL‐23 form a crucial bridge in Th17‐driven pathological processes.^[^ ^28^ ^]^ Existing studies have confirmed that Th17 differentiation requires TGF‐β combined with IL‐6, and IL‐23 stabilizes its function.^[^ ^48^, ^49^ ^]^ However, they have not clarified how NAFLD specifically drives the metabolic mechanisms of this cytokine environment in the kidneys. Therefore, this study elucidated the core pathological logic underlying the exacerbation of renal fibrosis in NAFLD. Hepatic ANGPTL8 activates renal macrophages through an ALOX5AP‐dependent pathway, amplifying IL‐6/IL‐23 signaling, and intensifying the Th17 response.

Thus, the discovery of this axis has significant therapeutic implications. While CCR2 inhibitors (such as RS504393) can alleviate general renal fibrosis, their effectiveness in NAFLD‐induced pathologies remains uncertain.^[^ ^50^, ^51^ ^]^ In our NAFLD‐UUO model, CCR2 blockade significantly inhibited the abnormal expansion of CCR2^+^PIRB^+^ macrophages, reducing IL‐6 and IL‐23 levels, Th17 cell proliferation, and the severity of fibrosis. Unlike previous studies that focused solely on monocyte infiltration, we identified and targeted a specific CCR2^+^ subset (PIRB^+^) activated by NAFLD. In vitro coculture experiments further confirmed that ANGPTL8 secreted by hepatocytes was both necessary and sufficient to initiate macrophage‐renal cell exclusion and inflammation via ALOX5AP induction in kidneys. Thus, targeting ANGPTL8/PIRB or ALOX5AP may offer greater specificity than broad‐spectrum CCR2 antagonists, avoiding systemic immune suppression.

Our study had some limitations. While we have identified ANGPTL8‐PIRB binding as an upstream triggering signal and established a link between ALOX5AP and cytokine secretion, direct intracellular signaling cascades (e.g., whether PIRB activates NF‐κB/STAT3 via ALOX5AP metabolites) remain unexplored. ALOX5AP generates various eicosanoids, such as leukotrienes and HETEs, with complex immune functions (e.g., leukotrienes, HETEs). Future lipidomic studies should clarify the metabolites that drive IL‐6/IL‐23 production in CCR2^+^PIRB^+^ macrophages. For human sample validation, analyzing the correlation between serum ANGPTL8 levels and renal CCR2^+^PIRB^+^ macrophage infiltration and fibrosis in patients is crucial for translational applications.

This study enhances our understanding of the novel mechanism by which the liver drives the renal immune‐metabolic imbalance. The ANGPTL8/PIRB/ALOX5AP axis is an interventional mechanism in the NAFLD‐CKD epidemiology. Potential strategies include: 1) developing anti‐ANGPTL8 monoclonal antibodies or PIRB blockers to intercept the liver‐kidney axis; 2) repurposing 5‐LOX/FLAP inhibitors (e.g., zileuton) for macrophage‐specific inflammatory treatment of CKD; and 3) using serum ANGPTL8 as a novel biomarker for risk stratification of CKD progression in NAFLD patients. Our study elucidates the organ interaction mechanism at the molecular, cellular, and metabolic levels, opening new avenues for precise intervention in fibrosis exacerbated by metabolic diseases.

## 4 Conclusion 

Our findings indicate that the ANGPTL8/PIRB/ALOX5AP axis is a crucial signaling pathway for liver‐kidney interactions. Elevated ANGPTL8 levels released by fatty liver cells circulate in the bloodstream and interact with CCR2^+^PIRB^+^ macrophages in the kidneys. This interaction regulates arachidonic acid metabolism within macrophages through the LOX‐5 (ALOX5) and FLAP (ALOX5AP) protein complexes, leading to their activation. Activated macrophages then release inflammatory factors that modulate Th17 cell responses, facilitating the recruitment and activation of additional proinflammatory immune cells at the damaged sites, and may contribute to the progression of renal fibrosis by enhancing the activity of TGF‐β.^[^ ^46^, ^47^ ^]^ Therefore, this mechanism may serve as a novel therapeutic target for emerging drugs to reduce the risk of renal fibrosis in patients with NAFLD.

## 5 Experimental Section

### Establishment of NAFLD Animal Models

NAFLD models were established using 6‐week‐old male C57BL/6 mice obtained from Zhaoqing Huaxia Kaiqi Biotechnology Co., Ltd. All mice had a C57BL/6J genetic background and were housed in a specific pathogen‐free environment.

Mice in the NAFLD group were subjected to a 12‐week feeding regimen with the D12492 research diet (Dyets HF60, Daitz Biotechnology Co., Ltd.) to induce mouse NAFLD modeling, while animals in the control group were fed a normal diet (1 060 012, Jiangsu Xietong Pharmaceutical Bio‐engineering Co., Ltd.). For the db/db mouse‐induced NAFLD model, db/db mice were fed a standard diet for 12 weeks to induce the NAFLD model, while the control db/m mice were also fed a standard diet for 12 weeks. Throughout the experimental period, the mice in both groups were weighed weekly. At the conclusion of the modeling period, all mice were euthanized, and serum samples were collected for liver function and lipid tests. Liver tissues were extracted from the animals for visual inspection, followed by fixation with 4% paraformaldehyde for 24 hours, dehydration, and embedding. The Animal Ethics Committee of Southern Medical University Shenzhen Hospital approved all animal experiments (project number:No.2024‐0315). Approximately 100 mice were utilized for the relevant studies, and each protocol adhered to the guidelines for the care and use of laboratory animals. NAFLD Model Established in 6‐Week‐Old Male C57BL/6 Mice

### Establishment of UUO Animal Models

CD and HFD mice were subjected to unilateral ureteral ligation respectively: 1) regular diet unilateral ureteral ligation mice (CD‐U), regular diet own control (CD‐Ctrl); 2) high‐fat diet unilateral ureteral ligation mice (HFD‐U), high‐fat diet own control (HFD‐Ctrl); (3) CCR2 inhibitor (RS504393) treated HF+UUO mice (AHD‐U), PEG400 vector‐treated as control mice. Mice were injected intraperitoneally with pentobarbital under general anesthesia to establish UUO models. The left ureter was exposed through a left‐sided incision and ligated with a 4‐0 wire, which was cut between 2 ligature points. Self‐controls were not ligated. In an in vivo CCR2 inhibition experiment, RS504393 was dissolved in PEG400 and administered orally immediately after UUO at a dose of 2 mg/kg twice daily for 7 days. Both groups received oral gavage using disposable feeding needles and syringes. On the 7th day after surgery, the mice were euthanized, and both kidneys were removed for further analysis. All experimental procedures were conducted in accordance with the guidelines approved by the Animal Protection and Utilization Committee of Southern Medical University Shenzhen Hospital.

### Establishment of AAV Virus‐Treated Animal Model

The ALOX5AP monocyte‐macrophage specific knockout model was established using recombinant AAV6 (Adeno‐Associated Virus Serotype 6). Six‐week‐old male C57BL/6 mice were fed a high‐fat diet for 7 weeks. Afterward, the mice were tail‐injected with AAV‐LYSM‐ALOX5AP virus and AAV‐LYSM‐Control virus. After 28 days of viral treatment, the mice underwent UUO surgery. The experimental group was treated with AAV6‐U6>{mAlox5ap‐SagRNA}>Kozak‐SaCas9, while the control group was treated with AAV6‐CMV>Kozak‐EGFP. The sgRNA sequence targeting the ALOX5AP gene was 5′‐GACCGGGACTCTTGCCTTTGA‐3′.

### Seglen Two‐Step Perfusion Method for Isolating Primary Hepatocytes from mice

Male mice aged 6‐8 weeks were subjected to a 5‐minute perfusion with Hanks' balanced salt solution via the inferior vena cava. After the liver color changed to beige or light brown, 0.5 mg mL^−1^ collagenase IV (LS004189, Worthington Biochem) was perfused into the liver, with all perfusion solutions maintained at 42 °C in a water bath. Upon observing a honeycomb‐like appearance on the liver surface, perfusion was immediately stopped, and the liver was excised. The digested liver cells were isolated using ice‐cold DMEM containing 10% FBS and 2% penicillin/streptomycin, filtered through a 100 µm cell strainer, and centrifuged at 50 × g for 5 minutes at 4 °C. The cell pellet was washed twice with DMEM containing 10% FBS, then cultured on plates coated with mouse tail collagen I (354236, Corning). Cells were maintained in DMEM supplemented with 10% FBS and 1% penicillin/streptomycin, and functional studies commenced the following day.

### In Vitro Induction of NAFLD Model

Palmitic acid (57‐10‐3, TargetMol) was dissolved in anhydrous ethanol at 70 °C to prepare a 500 × 10^−3^ m stock solution. Oleic acid (112‐80‐1, TargetMol) was dissolved in DMSO to prepare a 1000 mM stock solution. A complete culture medium containing free fatty acids was prepared with a ratio of palmitic acid to oleic acid of 1:2, incubated at 55 °C until clarified, and then sterilized by filtration before use. For AML12 cells, the final concentration of palmitic acid was 0.5 mM and oleic acid was 1 mM, using DMEM/F12 medium supplemented with 10% FBS and 1% penicillin/streptomycin, and incubated at 37 °C in a 5% CO2 incubator for 24 hours. For primary hepatocytes, the final concentration of palmitic acid was 0.25 mM and oleic acid was 0.5 mM, using DMEM supplemented with 10% FBS and 1% penicillin/streptomycin, and incubated at 37 °C in a 5% CO2 incubator for 24 hours.

### Plasmid Transfection and Lentiviral Transduction in Mouse Macrophage Cell Line RAW264.7

Plasmids targeting ALOX5AP with shRNA sequences were synthesized by Guangzhou AikeBiotechnology Co., Ltd. (Guangzhou, China). Briefly, the sh‐RNA sequences targeting ALOX5AP were cloned into the pLKO.1‐puro vector. The sequences are as follows: (shRNA#1, 5′‐ccggGCATGAAAGCAAGGCGCATAACTCGAGTTATGCGCCTTGCTTTCATGCTTTTTGAATT‐3′; shRNA#2, 5′‐ccggGCTAGCACCAGCCTGGTTGTCCTCGAGGACAACCAGGCTGGTGCTAGCTTTTTGAATT‐3′; and the scrambled control sequence, 5′‐TCCTAAGGTTAAGTCGCCCTCG‐3′); Transfection was performed in 293T cells using Hieff Trans Liposome DNA Transfection Reagent (40802ES08, YEASEN) according to the manufacturer's instructions, and lentiviral particles were collected from the cell culture medium.

The mouse macrophage cell line RAW264.7 come from Guangzhou First People's Hospital. RAW264.7 cells were transduced with lentiviral particles for 24 hours, after which the lentivirus‐containing medium was removed and replaced with DMEM supplemented with 10% FBS. Cells were then selected with puromycin (5 µg mL^−1^, P8230,Solarbio) for 1 week, and maintenance was continued with 3 µg mL^−1^ puromycin.

### Induction of BMDMs and Establishment of Liver Co‐Culture Model

Bone marrow cells from mice were cultured with macrophage colony‐stimulating factor (M‐CSF) for one week to induce BMDMs. The day before the experiment, BMDMs were seeded in the lower chamber of a 12‐well Transwell plate (3460, Corning) to allow adherence, while PMH were seeded in the upper chamber. Cells were then cultured with 500 ng mL^−1^ recombinant ANGPTL8 (rANGPTL8) (HY‐P700561, MCE) or 10 µg mL^−1^ anti‐PIRB neutralizing antibody (MA5‐24049, ThermoFisher), or both treatments combined. After incubation, flow cytometry was used to analyze the expression of ALOX5AP in BMDM cells and the mRNA expression of inflammatory factors.

### In Vitro Polarization of Mouse Th17 Cells

Cells were isolated from the spleen of mice and CD4^+^ T cells were purified using flow cytometry. The isolated T cells were suspended in RPMI 1640 medium supplemented with 10% fetal bovine serum (FBS), 1% L‐glutamine, and 1% penicillin/streptomycin. T cells were seeded in 24‐well or 96‐well plates at a density of 2×10^5 cells per well. Cells were activated using anti‐CD3 antibody (1 µg mL^−1^) and anti‐CD28 antibody (1 µg mL^−1^), and cytokines were added to induce Th17 cell differentiation: IL‐6 (20 ng mL^−1^), TGF‐β (5 ng mL^−1^), IL‐23 (20 ng mL^−1^), anti‐IFN‐γ antibody (1 µg mL^−1^) to inhibit IFN‐γ secretion, and anti‐IL‐4 antibody (1 µg mL^−1^) to inhibit IL‐4 secretion. The cells were cultured for 72 hours in a 37 °C, 5% CO2 incubator. Th17 cell differentiation was assessed using anti‐IL‐17A antibody.

### Extraction of Mouse Renal Fibroblasts

Mouse kidneys were cut into small pieces and digested in RPMI 1640 containing collagenase IV (LS004189, Worthington‐Biochem) at 37 °C. After digestion, the cell suspension was filtered through a 70 µm cell strainer to remove undigested tissue chunks. The filtered cell suspension was transferred to a centrifuge tube and washed with an adequate amount of PBS. Centrifugation was performed at 300–400 × *g* for 5–10 min to remove the supernatant. The washing step was repeated to ensure the removal of most extracellular matrix and residual digestion enzymes. The cell pellet was resuspended in DMEM culture medium (containing 10% FBS and 1% penicillin‐streptomycin) and seeded in culture dishes. Cells were cultured in a 37 °C, 5% CO2 incubator. Fibroblasts were selectively cultured by changing the medium every 2‐3 days. After one week, cells were subjected to morphological assessment and immunofluorescence staining for identification.

### Histology

Mice were euthanized, and following cardiac perfusion, fresh tissues were harvested and fixed in 4% paraformaldehyde. Subsequently, the tissues were embedded in paraffin and sectioned to a thickness of 4 µm. The sections were then oven‐baked at 60 °C.

### Masson Staining

Paraffin sections were deparaffinized to water and used Servicebio Masson Kit (G1006‐20ML,Servicebio). Then immersed it in Servicebio Masson A liquid overnight, followed by rinsing under running tap water. The sections were subsequently immersed into an equal mixture of Servicebio Masson B and Servicebio Masson C, and then washed and differentiated. Afterward, the sections were immersed into Servicebio Masson D solution for 6 minutes and rinsed under tap water. They were then stained in Servicebio Masson E solution for 1 minute, followed by immediate transfer into Masson F solution for 2‐30 seconds. The sections were rinsed again and differentiated with 1% acetic acid, dehydrated in anhydrous ethanol, made transparent with xylene, and finally sealed before observation under a microscope and scanning with the KFBIO KF‐FL‐020 scanning system.

### Oil Red Dyeing

The paraffin sections were deparaffinized to water and used Servicebio oil red Kit (G1015,Servicebio), followed by immersion into oil red O staining solution for 8‐10 minutes (covered and protected from light). Afterward, the sections were removed, allowed to stay for 3 seconds, and then sequentially immersed in two vats of 60% isopropyl alcohol for differentiation. Subsequently, the sections were dipped and washed in pure water, removed, allowed to stay for 3 seconds, and then immersed in hematoxylin restaining solution. After dipping and washing in pure water for differentiation and returning to blue, the staining effect was microscopically examined. The slices were sealed with glycerol gelatin sealing agent and observed under the microscope, followed by scanning with the KFBIO KF‐FL‐020 scanning system.

### HE Staining

The paraffin sections underwent deparaffinization to water, followed by staining with hematoxylin staining solution for 3‐5 minutes. After staining, they were rinsed in tap water, subjected to differentiation in a differentiation solution, and washed again in tap water. Next, the sections were subjected to a process to return them to blue using a return blue solution, followed by rinsing in running water. Subsequently, they were dehydrated in 95% alcohol for 1 minute and stained with eosin stain for 15 seconds. Dehydration was achieved using anhydrous ethanol, followed by clearing in xylene and sealing. Finally, the sections were observed under a microscope and scanned using the KFBIO KF‐FL‐020 scanning system.

### Immunohistochemistry

The paraffin sections were initially deparaffinized in water, followed by antigen retrieval in a sodium citrate solution. Endogenous peroxidase activity was blocked by immersion in a 3% hydrogen peroxide solution. Subsequently, the sections were treated with 5% BSA for blocking, after which they were incubated overnight at 4 °C in a humidified chamber with the primary antibody. Following this, the sections were exposed to an HRP‐labeled secondary antibody. Color development was controlled using freshly prepared DAB chromogenic solution, with the reaction being terminated upon reaching the desired intensity through rinsing with tap water. Hematoxylin was applied to restain the cell nuclei, and a differentiation solution was briefly employed to enhance nuclear contrast. Subsequently, a blue return solution was utilized to restore the blue color of the nuclei, followed by rinsing with running water. The sections were dehydrated in anhydrous ethanol, cleared in xylene, sealed, and subjected to microscopic observation and scanning using the PRECICE 500B scanning system.

### Immunohistochemistry (IHC)

Paraffin sections were deparaffinized and hydrated before performing antigen retrieval in sodium citrate or EDTA solution. Endogenous peroxidase was blocked by immersing the sections in 3% hydrogen peroxide solution. Blocking was done using 5% BSA, followed by incubation with primary antibodies, including anti‐α‐SMA (ab5694, Abcam), anti‐ANGPTL8 (EM2001‐08, HUABIO), anti‐ LILRB2 (CSB‐PA013000LA01HU, CUSABIO), and anti‐ALOX5AP (ab314652, Abcam), in a humid chamber at 4 °C overnight. After washing with PBS, the sections were incubated with HRP‐conjugated goat anti‐rabbit (CW0103S, CWBIO) and HRP‐conjugated goat anti‐mouse (CW0102S, CWBIO) secondary antibodies. Freshly prepared DAB substrate (ZLI‐9017, ZSGB‐BIO) was used for chromogenic development under a microscope to control the reaction time. The color reaction was stopped by rinsing with tap water. Hematoxylin was used to counterstain the nuclei, followed by differentiation for a few seconds, bluing, and washing with running water. The sections were dehydrated with absolute ethanol, cleared in xylene, and mounted. Observations were made under a microscope, and the slides were scanned using the KFBIO KF‐FL‐020 scanning system.

### Immunofluorescence

Paraffin sections were deparaffinized and hydrated, followed by antigen retrieval using sodium citrate or EDTA solution. Blocking was performed with 5% BSA. Sequential immunofluorescence was carried out by adding primary antibodies, including anti‐CCR2 (ET1611‐65, HUABIO), anti‐LILRB2 (CSB‐PA013000LA01HU, CUSABIO), anti‐CD68 (HA601115, HUABIO), and anti‐ALOX5AP (ab314652, Abcam), and incubating them overnight at 4 °C in a humid chamber. FITC‐conjugated secondary antibodies (GB22303, Servicebio), Cy3‐conjugated secondary antibodies (GB21301, Servicebio), Alexa Fluor 594‐conjugated secondary antibodies (GB28301, Servicebio), and Alexa Fluor 647‐conjugated secondary antibodies (A0468, Beyotime) were then added. DAPI was used for nuclear counterstaining. Anti‐fade mounting medium was applied to prevent quenching, and images were captured using a Zeiss LSM880 laser confocal microscope. The slides were scanned using the KFBIO KF‐FL‐020 scanning system.

### Flow Analysis and Sorting—Preparation of Single Cell Suspension

The kidneys were finely minced and subjected to oscillation digestion in RPMI 1640 supplemented with collagenase(LS004189, Worthington‐biochem) for 2 hours at 37 °C. Following digestion, the suspension was filtered through a 70 µm sieve to eliminate undigested aggregates and debris. Erythrocytes were completely lysed using erythrocyte lysate, followed by centrifugation at 400 RCF to discard the supernatant and retain the cellular pellet, from which the total cell count was enumerated. The isolated cells from this process will be utilized for FACS analysis. Subsequently, cells will be resuspended in 10% FBS RPMI 1640 complete medium supplemented with Brefeldin A (50504ES08, YEASEN), phorbol 12‐myristate 13‐acetate (50601ES, YEASEN), and ionomycin (56092‐82‐1), YEASEN, and incubated at 37 °C for 5 hours to facilitate intracellular cytokine staining.

### Flow Cytometry

Anti‐mouse CD 16/32 (101302, BioLegend) was incubated with the collected cells for 30 minutes at 4 °C to prevent nonspecific binding. For intracellular staining, cells were treated with a fixed membrane‐breaking reagent (00‐5523‐00, ThermoFisher) and labeled with suitable extracellular antibodies before analysis. Perform flow cytometry using BD LSRFortessa X‐20 or BD FACSVerse instruments, sort primary cells with the BD Aria III, and analyze the data with FlowJo software.

The direct staining flow cytometry employs the following antibodies:

Fixable Viability Dye (65‐0863‐14, ThermoFisher), CD45 (552 848&550 993, BD), CD11b (550 993&557 396, BD), F4/80 (565 410, BD), CCR2 (150 637, BD), CD3 (100 235, BioLegend), CD4 (100 559, BioLegend), IL‐17A (506 904, BioLegend), IFNγ (505 849, BioLegend), IL‐13 (159 407, BD), and FOXP3 (126 404, BioLegend).

The indirect staining flow cytometry utilizes the following antibodies:

Anti‐PIRB (MA5‐24049, ThermoFisher) primary antibody, anti‐ALOX5AP (ab314652, Abcam) primary antibody, FITC‐labeled goat anti‐rat IgG (GB22302, Servicebio), and FITC‐labeled goat anti‐rabbit IgG (GB22303, Servicebio).

### EdU Assay for Cell Proliferation

Mouse renal fibroblasts were cultured under different treatments for 72 hours, followed by the addition of 10 × 10^−6^ m EdU (C0081S, Beyotime) to label the cells for 2 hours at 37 °C. After PBS washing, the cells were collected via trypsin digestion, fixed in 4% paraformaldehyde for 15 minutes, and permeabilized with PBS containing 0.3% Triton X‐100. The cells were then fluorescently labeled with Alexa Fluor 647 through a click chemistry reaction, which specifically binds to EdU. Data were analyzed using a BD FACSVerse instrument and FlowJo software, and the proportions of EdU^+^ (S‐phase proliferating cells) and EdU‐ cells were calculated.

### RNA Isolation and Real‐Time Quantitative PCR (RT─qPCR)

The extraction of total RNA from kidney tissue was carried out utilizing EZ‐Press RNA Purification Kit (B0004D, EZBioscience), followed by reverse transcription using the HiFiScript RNA Reverse Transcription Kit with gDNA Remove (CW2020M, CWBIO). Real‐time quantitative PCR was conducted employing the Q7 RT‐PCR Detection System and SYBR premixed Ex Taq (CW3008H, CWBIO). Relative gene expression levels were determined utilizing actin (mouse β‐actin) as an internal reference control and quantified via the ΔΔCT method, with the results normalized to the relative expression compared to mouse β‐actin. Primer sequences utilized in the experiments are delineated in Table S2 (Supporting Information).

### Coimmunoprecipitation (Co‐IP)

Add Co‐IP cell lysis buffer (P0013, Beyotime) to BMDM cells, lyse on ice for 20 minutes, and collect the cell supernatant after centrifugation. Mix the obtained supernatant with Anti‐his Tag (AF2876, Beyotime), anti‐Angptl8 (ab180915, abcam) or anti‐PIRB (MA5‐24049, ThermoFisher), incubate overnight on a shaker at 4 °C, and then incubate with Protein A/G Magnetic Beads (HY‐K0202, MedChem Express) at 4 °C for 2 hours. After washing 3 times with co‐IP lysis buffer, boil the protein samples in 1×SDS loading buffer, and further analyze the immunoprecipitated proteins using Western blotting.

### Western Blot

Renal tissues underwent a precooled PBS rinse preceding homogenization. The homogenized specimens were subsequently subjected to lysis in RIPA buffer (P0013F, Beyotime), supplemented with a protease inhibitor cocktail (4693116001, Roche Diagnostics), a phosphatase inhibitor mixture (4906845001, Roche Diagnostics), and phenylmethylsulfonyl fluoride (PMSF) (ST506, Beyotime). Following a 30‐minute incubation in an ice bath, the lysates were subjected to centrifugation at 12,000g for 30 minutes, and the resultant supernatant was preserved at –80 °C. The total protein concentration was assessed utilizing the BCA Protein Assay Kit (23 225, Thermo Fisher Scientific). Subsequently, protein samples (10‐20 µg) were diluted with a 5‐fold excess of sample loading buffer, subjected to a 10‐minute heat treatment in a metal bath, separated via SDS polyacrylamide gel electrophoresis (SDS‐PAGE), and then transferred onto a PVDF membrane for antibody detection. Immunoreactive proteins were discerned using an enhanced chemiluminescence detection system.

A variety of primary antibodies were employed, encompassing Anti‐αSMA (ab5694, abcam), Anti‐Fibronectin (ab2413, abcam), Anti‐Flap (ab314652, abcam), Anti‐GAPDH (CW0100M, CWBIO), Anti‐his (AF2876, Beyotime), Anti‐ANGPTL8 (ab180915, abcam) and Anti‐PIRB (MA5‐24049, ThermoFisher). Secondary antibodies utilized included HRP‐conjugated goat anti‐mouse IgG (H+L)(CW0102S, CWBIO) and HRP‐conjugated goat anti‐rabbit IgG (H+L) (CW0103S, CWBIO). This protein blotting methodology was tailored for the detection of specific protein expression levels in kidney samples through the targeted antibodies utilized.

### Molecular Docking

The amino acid sequences of ANGPTL8 and PIRB were obtained from UniProt (https://www.uniprot.org/), and the protein crystal structures were predicted using AlphaFold3. The resulting protein structures were processed using the Protein Preparation Wizard module in Schrödinger software. Following this, protein‐protein interaction simulations were conducted using the protein‐protein docking module, with the “Number of ligand rotations to probe” set to 70000 and “Maximum poses to return” set to 30. A lower interaction score indicates a more favorable binding free energy between the ligand and receptor, suggesting higher binding stability. The protein‐protein interaction complex with the lowest interaction score was visualized, with different chains labeled in distinct colors and the surface displayed to show a 3D view. Additionally, the Protein Interaction Analysis module was employed to determine the specific binding regions between ANGPTL8 and PIRB.

### Enzyme‐Linked Immunosorbent Assay (ELISA)

ELISA assays were conducted to assess the levels of cytokines such as IL‐21, IL‐23, and IL‐6 in the supernatant of kidney tissue. To detect cytokines within the kidney tissues, samples were washed, weighed at 10 mg, and finely sectioned into small fragments, followed by addition of 100 µL of PBS. Subsequently, the mixture was homogenized using a homogenizer, and after centrifugation, the supernatant was collected for analysis. All experimental procedures were carried out in accordance with the respective manufacturer's protocols for the appropriate ELISA kits: Mouse IL‐21 ELISA Kit (E‐EL‐M1273, Elabscience), Mouse IL‐23 ELISA Kit (E‐MSEL‐M0037, Elabscience), Mouse IL‐6 ELISA Kit (555240, BD).

### Mendelian Randomization Analysis

Bidirectional Mendelian Randomization (MR) analysis employs genetic variants of single nucleotide polymorphisms (SNPs) as instrumental variables (IVs) to elucidate the causal relationship between NAFLD and CKD, considering each as both exposure and outcome. NAFLD data are sourced from the GWAS Catalog database (GCST90091033), which includes 1106 cases and 8571 controls. CKD data are obtained from the IEU OpenGWAS project website, designated as “ieu‐a‐1102,” encompassing 12385 cases and 104780 controls. The primary analysis utilizes the inverse variance weighted (IVW) method, supplemented by the weighted median estimator (WME), MR‐Egger regression, simple models, and weighted models, with SNPs as instrumental variables to explore the causal relationship between NAFLD and CKD. Cochran's Q test assesses statistical heterogeneity among SNPs, while MR‐Egger regression's intercept and MR_pleiotropy_test examine the horizontal pleiotropy of SNPs. Leave‐one‐out analysis evaluates the influence of individual SNPs on IVW results, and funnel plots are used to assess potential bias in SNPs. In the positive causal inference of NAFLD on CKD, the IVW analysis showed: OR = 1.91, 95% CI: 1.63–2.23, P < 0.05. The results of MR‐Egger regression, WME, and WM were consistent with those of the IVW method, with the overall effect direction aligning. The findings are robust, indicating a positive causal relationship between genetically predicted NAFLD and CKD, suggesting that NAFLD may increase the risk of renal fibrosis. The Cochrane's Q test indicated no heterogeneity in the data (P > 0.05), and the MR‐Egger intercept did not provide substantial evidence of directional pleiotropy in NAFLD (P > 0.05). As this study exclusively utilizes publicly available data, no specific ethical approval was required. Comprehensive ethical approval and informed consent details for each GWAS dataset are appropriately documented in their respective original publications.

### Single‐Cell RNA‐seq

Following left ventricular infusion of PBS in mice, kidney single‐cell suspensions were prepared and processed using the 10X Chromium single‐cell instrument. Upon completion of gene expression profiling by “ Cell Ranger ”, the expression matrix was transferred to “ Seurat ” for subsequent analysis. “ DoubletFinder ” was employed to calculate the probability of GEMs being doublets, and based on the relationship between the number of valid cells provided by 10X and the doublet rate, the doublet rate of each sample was computed and subsequently filtered. “ Harmony ” was utilized for data integration and batch effect correction, while “ singleR ” was employed for automated annotation of all cells. To construct a single‐cell pseudotime trajectory analysis and identify genes undergoing changes during cellular transitions, the “ Monocle2 (v.2.4.0) ” algorithm was applied to CCR2^+^ macrophages. The “ differentialGeneTest ” function in Monocle2 was used to identify highly variable genes along the pseudotime trajectory, with a q‐value threshold set at <0.01. Gene changes at different branching points were analyzed using the “ BEAM ” function. The R package “ cellchat ” was utilized for analysis of the intensity and quantity of immune cell communication.

### Untargeted Metabolomics

Untargeted metabolomics analysis involves processing raw data to acquire precursor molecules under positive and negative ion modes, normalizing quantitative results, and finally identifying and quantifying the data. All metabolites are subjected to z‐score normalization using the R package pheatmap (v1.0.12), followed by clustering analysis and heatmap visualization. Differential metabolites between various comparison groups are selected based on the Variable Importance in Projection (VIP) values from Orthogonal Projections to Latent Structures Discriminant Analysis (OPLS‐DA) and the P values from univariate statistical analysis, specifically T‐tests (where a threshold for significance is set at OPLS‐DA VIP ≥ 1 and T‐test P < 0.05). Furthermore, volcano plots of differential metabolites are generated based on VIP values and P values. Qualitative results of metabolites are utilized to retrieve corresponding compound IDs (C_id) from the KEGG database for KEGG pathway enrichment analysis and Metpa topological analysis. Perform a joint correlation analysis of transcriptomic and metabolomic data using the R package “Hmisc,” and visualize the results as a correlation heatmap utilizing the “pheatmap” package. Genes related to the linoleic acid pathway are derived from the MSigDB database (https://www.gsea‐msigdb.org/gsea/msigdb/).

### Human Renal Paraffin‐Embedded Sections

Renal paraffin‐embedded sections were obtained from the Department of Nephrology at Renmin Hospital, Longhua District, Shenzhen, Southern Medical University. These sections were derived from samples collected after renal fine‐needle aspiration, and all patients from whom the samples were obtained had CKD with renal fibrosis. The samples were randomly selected based on gender and age and were categorized into experimental (CKD+NAFLD) and control (CKD) groups according to imaging and liver function tests for the presence of NAFLD. All samples were used for immunohistochemistry and immunofluorescence staining, and publication consent was obtained. The human research was approved by the Ethics Committee of Southern Medical University Shenzhen Hospital (project number: NYSZYYEC20191209) and conducted in accordance with the principles of the Helsinki Declaration. Written informed consent was obtained from all patients.

### Statistical Analysis

Statistical analyses were conducted utilizing GraphPad Prism 8. All data are presented as mean ± SEM, unless otherwise specified. For comparisons involving three or more groups, the one‐way or two‐way ANOVA with Turkey's multiple comparisons test was applied to compare individual groups. For comparisons between the two groups, an unpaired t‐test was utilized. Correlation analysis was performed using Pearson's correlation coefficient. Statistical significance was defined as *P* < 0.05. The data are depicted as mean ± Standard Error of Mean(SEM). Detailed statistical parameters and the number of mice utilized in each experiment are provided in the figure legends. Statistical significance levels are denoted as follows: *P < 0.05; **P < 0.01; ***P < 0.001; ****P < 0.0001.

## Conflict of Interest

The authors declare no conflict of interest.

## Author Contributions

Contributors DLS conceived the study and obtained funding. JG and GJQ conducted the analysis of single‐cell sequencing and revised the manuscript, while SQW, QZL, JFL, and XYL analyzed the metabolomic sequencing data and drafted the manuscript. SQW was responsible for the specific experimental design and completed the initial draft of the manuscript. DLS, JG and GJQ revised the manuscript. DWS, SYH, and YSW performed experiments and contributed to data analysis. LW and WM contributed to the collection of clinical pathology specimens. QZL, YYZ, LTW, HL, JCC, YXC, HYZ and YQZ assisted with experimental procedures. S.W., D.S., S.H. and Y.W. contributed equally to this work.

## Data and Materials Availability

The data sets involved in this study include GEO database—GSE76882 and GSE89632, KEGG database (https://www.kegg.jp/), MSigDB database (https://www.gsea‐msigdb.org/gsea/msigdb/). Our single‐cell transcriptome sequencing data and nontargeted metabolomics sequencing have been uploaded to the NCBI database (GSE281485) and OMIX database (OMIX007604). The original contributions presented in the study are included in the article/Supplementary Material. Further data will be shared after approval of the proposal by the corresponding author and a signed data access agreement

## Supporting information



Supporting Information

Supplemental Table 1

Supplemental Table 2

## Data Availability

The data that support the findings of this study are available from the corresponding author upon reasonable request.

## References

[1] Kidney Disease: Improving Global Outcomes (KDIGO) CKD Work Group , Kidney Int. 2024, 105, S117.38490803

[2] B. D. Humphreys , Annu. Rev. Physiol. 2018, 80. 10.1146/annurev-physiol-022516-034227 29068765

[3] Q. Yuan , B. Tang , C. Zhang , Signal. Transduct. Target Ther. 2022, 7, 182. 35680856 10.1038/s41392-022-01036-5 PMC9184651

[4] M. Ruiz‐Ortega , S. Rayego‐Mateos , S. Lamas , A. Ortiz , R. R. Rodrigues‐Diez , Nat. Rev. Nephrol. 2020, 16, 269. 32060481 10.1038/s41581-019-0248-y

[5] M. Ruiz‐Ortega , S. Lamas , A. Ortiz , Am. J. Kidney Diseases 2022, 80, 251. 34999158 10.1053/j.ajkd.2021.11.010

[6] K. Yau , R. Kuah , D. Cherney , T. Lam , Nat. Rev. Endocrinol. 2024, 20, 321. 38351406 10.1038/s41574-024-00951-7

[7] Q. Qian , M. Li , Z. Zhang , S. W. Davis , K. Rahmouni , A. W. Norris , H. Cao , W.‐X. Ding , G. S. Hotamisligil , L. Yang , Cell Metab. 2024, 36, 1550. 38718793 10.1016/j.cmet.2024.04.014 PMC11222033

[8] R. Manikat , M. H. Nguyen , Clin. Mol. Hepatol. 2023, 29, s86. 36603574 10.3350/cmh.2022.0442 PMC10029963

[9] D. Raj , B. Tomar , A. Lahiri , S. R. Mulay , Pharmacol. Res. 2020, 152, 104617. 31881272 10.1016/j.phrs.2019.104617

[10] T.‐Y. Wang , R.‐F. Wang , Z.‐Y. Bu , G. Targher , C. D. Byrne , D.‐Q. Sun , M.‐H. Zheng , Nat. Rev. Nephrol. 2022, 18, 259. 35013596 10.1038/s41581-021-00519-y

[11] D.‐Q. Sun , Y. Jin , T.‐Y. Wang , K. I. Zheng , R. S. Rios , H.‐Y. Zhang , G. Targher , C. D. Byrne , W.‐J. Yuan , M.‐H. Zheng , Metab.‐Clin. Exp. 2021, 115, 154433. 33212070 10.1016/j.metabol.2020.154433

[12] G. Musso , R. Gambino , J. H. Tabibian , M. Ekstedt , S. Kechagias , M. Hamaguchi , R. Hultcrantz , H. Hagström , S. K. Yoon , P. Charatcharoenwitthaya , J. George , F. Barrera , S. Hafliðadóttir , E. S. Björnsson , M. J. Armstrong , L. J. Hopkins , X. Gao , S. Francque , A. Verrijken , Y. Yilmaz , K. D. Lindor , M. Charlton , R. Haring , M. M. Lerch , R. Rettig , H. Völzke , S. Ryu , G. Li , L. L. Wong , M. Machado , et al., PLoS Med. 2014, 11, 1001680. 10.1371/journal.pmed.1001680 PMC4106719 25050550

[13] Y. Takahashi , T. Fukusato , World J. Gastroenterol. 2014, 20, 15539. 25400438 10.3748/wjg.v20.i42.15539 PMC4229519

[14] A. Mantovani , G. Petracca , G. Beatrice , A. Csermely , A. Lonardo , J. M. Schattenberg , H. Tilg , C. D. Byrne , G. Targher , GUT 2022, 71, 156. 33303564 10.1136/gutjnl-2020-323082

[15] D. Q. Sun , F. Z. Ye , H. T. Kani , J. R. Yang , K. I. Zheng , H. Y. Zhang , G. Targher , C. D. Byrne , Y. P. Chen , W. J. Yuan , Y. Yilmaz , M. H. Zheng , Diab. Metab. 2020, 46, 288. 10.1016/j.diabet.2019.11.003 31786360

[16] J. Paik , P. Golabi , Z. Younoszai , A. Mishra , G. Trimble , Z. M. Younossi , Liver Int. 2019, 39, 342. 30347513 10.1111/liv.13992

[17] Y. Yilmaz , Y. O. Alahdab , O. Yonal , R. Kurt , A. E. Kedrah , C. A. Celikel , O. Ozdogan , D. Duman , N. Imeryuz , E. Avsar , C. Kalayci , Metab.‐Clin. Exp. 2010, 59, 1327. 20096896 10.1016/j.metabol.2009.12.012

[18] M. Caus , A. Eritja , M. Bozic , Int. J. Mol. Sci. 2021, 22, 11416. 34768854 10.3390/ijms222111416 PMC8583993

[19] M. Wang , Z. Wang , Y. Chen , Y. Dong , Int. J. Mol. Sci. 2022, 23, 747.35054932

[20] Z. Zhang , Y. Yuan , L. Hu , J. Tang , Z. Meng , L. Dai , Y. Gao , S. Ma , X. Wang , Y. Yuan , Q. Zhang , W. Cai , X. Ruan , X. Guo , J. Adv. Res. 2023, 47, 41. 36031141 10.1016/j.jare.2022.08.006 PMC10173191

[21] M. Abu‐Farha , A. Ghosh , I. Al‐Khairi , S. R. M. Madiraju , J. Abubaker , M. Prentki , Prog. Lipid Res. 2020, 80, 101067. 33011191 10.1016/j.plipres.2020.101067

[22] Y.‐H. Lee , S.‐G. Lee , C. J. Lee , S. H. Kim , Y.‐M. Song , M. R. Yoon , B. H. Jeon , J. H. Lee , B.‐W. Lee , E. S. Kang , H. C. Lee , B.‐S. Cha , Sci. Rep. 2016, 6, 24013. 27045862 10.1038/srep24013 PMC4820743

[23] D.‐P. Li , L. Huang , R.‐R. Kan , X.‐Y. Meng , S.‐Y. Wang , H.‐J. Zou , Y.‐M. Guo , P.‐Q. Luo , L.‐M. Pan , Y.‐X. Xiang , B.‐B. Mao , Y.‐Y. Xie , Z.‐H. Wang , M. Yang , R. He , Y. Yang , Z.‐L. Liu , J.‐H. Xie , D.‐L. Ma , B.‐P. Zhang , S.‐Y. Shao , X. Chen , S.‐M. Xu , W.‐T. He , W.‐J. Li , Y. Chen , X.‐F. Yu , Nat. Commun. 2023, 14, 4436. 37481670 10.1038/s41467-023-40183-3 PMC10363120

[24] P. J. Cunningham , P. F. Short , S. E. Feinleib , Med. Care 1995, 33, 432. 7731284 10.1097/00005650-199504000-00010

[25] E. K. Sean , P. Cockwell , Kidney Int. 2005, 68, 437. 16014021 10.1111/j.1523-1755.2005.00422.x

[26] S. C. Huen , L. G. Cantley , Annu. Rev. Physiol. 2017, 79, 449. 28192060 10.1146/annurev-physiol-022516-034219

[27] G. Pei , Y. Yao , Q. Yang , M. Wang , Y. Wang , J. Wu , P. Wang , Y. Li , F. Zhu , J. Yang , Y. Zhang , W. Yang , X. Deng , Z. Zhao , H. Zhu , S. Ge , M. Han , R. Zeng , G. Xu , Sci. Adv. 2019, 5, w5075. 10.1126/sciadv.aaw5075 PMC6594767 31249871

[28] B. R. Conway , E. D. O'Sullivan , C. Cairns , J. O'Sullivan , D. J. Simpson , A. Salzano , K. Connor , P. Ding , D. Humphries , K. Stewart , O. Teenan , R. Pius , N. C. Henderson , C. Bénézech , P. Ramachandran , D. Ferenbach , J. Hughes , T. Chandra , L. Denby , J. Am. Soc. Nephrol. 2020, 31, 2833. 32978267 10.1681/ASN.2020060806 PMC7790206

[29] J. Whelan , K. Fritsche , Adv. Nutr. 2013, 4, 311. 23674797 10.3945/an.113.003772 PMC3650500

[30] H. M. Kang , S. H. Ahn , P. Choi , Y.‐A. Ko , S. H. Han , F. Chinga , A. S. D. Park , J. Tao , K. Sharma , J. Pullman , E. P. Bottinger , I. J. Goldberg , K. Susztak , Nat. Med. 2015, 21, 37. 25419705 10.1038/nm.3762 PMC4444078

[31] A. E. Feldstein , R. Lopez , T. A.‐R. Tamimi , L. Yerian , Y.‐M. Chung , M. Berk , R. Zhang , T. M. McIntyre , S. L. Hazen , J. Lipid Res. 2010, 51, 3046. 20631297 10.1194/jlr.M007096 PMC2936759

[32] Y. Zhang , F. Song , M. Yang , C. Chen , J. Cui , M. Xing , Y. Dai , M. Li , Y. Cao , L. Lu , H. Zhu , Y. Liu , C. Ma , Q. Wei , H. Qin , J. Li , Adv. Sci. 2024, 11, 2306297. 10.1002/advs.202306297 PMC11132037 38477534

[33] R. Mashima , T. Okuyama , Redox Biol. 2015, 6, 297. 26298204 10.1016/j.redox.2015.08.006 PMC4556770

[34] J. U. An , S. E. Kim , D. K. Oh , Prog. Lipid Res. 2021, 83, 101110. 34144023 10.1016/j.plipres.2021.101110

[35] X. Cao , H. Guo , Y. Dai , G. Jiang , W. Liu , X. Li , D. Zhang , Y. Huang , X. Wang , H. Hua , J. Wang , K. Chen , C. Chi , H. Liu , Redox Biol. 2024, 71, 103096. 38387137 10.1016/j.redox.2024.103096 PMC10899062

[36] N. M. Isbel , P. A. Hill , R. Foti , W. Mu , L. A. Hurst , C. Stambe , H. Y. Lan , R. C. Atkins , D. J. Nikolic‐Paterson , Kidney Int. 2001, 60, 614. 11473644 10.1046/j.1523-1755.2001.060002614.x

[37] K. Hirahara , T. Nakayama , Int. Immunol. 2016, 28, 163. 26874355 10.1093/intimm/dxw006 PMC4889886

[38] S.‐Y. Zhang , Q.‐P. Xu , L.‐N. Shi , S.‐W. Li , W.‐H. Wang , Q.‐Q. Wang , L.‐X. Lu , H. Xiao , J.‐H. Wang , F.‐Y. Li , Y.‐M. Liang , S.‐T. Gong , H.‐R. Peng , Z. Zhang , H. Tang , Signal. Transduct. Target Ther. 2023, 8, 236. 37332010 10.1038/s41392-023-01438-z PMC10277282

[39] C. D. Mills , Front. Immunol. 2015, 6, 212. 25999950 10.3389/fimmu.2015.00212 PMC4419847

[40] D. G. Denardo , B. Ruffell , Nat. Rev. Immunol. 2019, 19, 369. 30718830 10.1038/s41577-019-0127-6 PMC7339861

[41] M. V. Nastase , J. Zeng‐Brouwers , J. Beckmann , C. Tredup , U. Christen , H. H. Radeke , M. Wygrecka , L. Schaefer , Matrix Biol. 2018, 68‐69, 293. 10.1016/j.matbio.2017.12.002 29253517

[42] Y. Sui , Q. Liu , C. Xu , K. Ganesan , Z. Ye , Y. Li , J. Wu , B. Du , F. Gao , C. Song , J. Chen , Cell Death Dis. 2024, 15, 67. 38238320 10.1038/s41419-023-06386-8 PMC10796330

[43] T. Kim , G. S. Vidal , M. Djurisic , C. M. William , M. E. Birnbaum , K. C. Garcia , B. T. Hyman , C. J. Shatz , Science 2013, 341, 1399. 24052308 10.1126/science.1242077 PMC3853120

[44] G. Zuo , L. Xuan , Z. Xin , Y. Xu , J. Lu , Y. Chen , M. Dai , D. Zhang , W. Wang , M. Li , Y. Bi , G. Ning , M. Xu , J. Clin. Endocrinol. Metab. 2021, 106, 3957. 10.1210/clinem/dgab425 34125886

[45] M.‐H. Hsieh , K.‐T. Wu , Y.‐Y. Chen , J.‐F. Yang , W.‐Y. Lin , N.‐C. Chang , C.‐Y. Lin , C.‐K. Huang , C.‐L. Wang , H.‐Y. Chuang , S.‐C. Lin , Y.‐K. Hsu , Y.‐S. Tsai , W.‐L. Chuang , M.‐L. Yu , C.‐Y. Dai , J. Formosan Med. Assoc. 2020, 119, 496. 31353118 10.1016/j.jfma.2019.07.007

[46] R. Zhang , Biochem. Biophys. Res. Commun. 2012, 424, 786. 22809513 10.1016/j.bbrc.2012.07.038

[47] S. Leung , X. Liu , L. Fang , X. Chen , T. Guo , J. Zhang , Cell Mol. Immunol. 2010, 7, 182. 20383174 10.1038/cmi.2010.22 PMC4002916

[48] T. Korn , E. Bettelli , M. Oukka , V. K. Kuchroo , Annu. Rev. Immunol. 2009, 27, 485. 19132915 10.1146/annurev.immunol.021908.132710

[49] J. M. Fletcher , R. Lonergan , L. Costelloe , K. Kinsella , B. Moran , C. O'Farrelly , N. Tubridy , K. H. G. Mills , J. Immunol. 2009, 183, 7602. 19917691 10.4049/jimmunol.0901881

[50] K. Kitagawa , T. Wada , K. Furuichi , H. Hashimoto , Y. Ishiwata , M. Asano , M. Takeya , W. A. Kuziel , K. Matsushima , N. Mukaida , H. Yokoyama , Am J Pathol 2004, 165, 237. 15215179 10.1016/S0002-9440(10)63292-0 PMC1618531

[51] D. Peroumal , P. S. Biswas , Annu. Rev. Immunol. 2024, 42, 35. 37906942 10.1146/annurev-immunol-052523-015141

