# Supplementary material for: Nonalcoholic Fatty Liver Disease Exacerbates the Advancement of Renal Fibrosis by Modulating Renal CCR2+PIRB+ Macrophages Through the ANGPTL8/PIRB/ALOX5AP Axis
Source: Adv Sci (Weinh). 2025 Sep 25;12(46):e09351. doi: 10.1002/advs.202509351 (PMC12697892; doi:10.1002/advs.202509351)
Supplement: Supplementary file 1 — Supporting Information [file ADVS-12-e09351-s001.docx]

Supplementary Materials for

**Nonalcoholic Fatty Liver Disease Exacerbates the Advancement of Renal Fibrosis by Modulating Renal CCR2^+^PIRB^+^ Macrophages through the ANGPTL8/PIRB/ALOX5AP axis**

**This file includes:**

Fig. S1. Results of the bidirectional MR analysis of NAFLD and CKD.

Fig. S2. Development of NAFLD Model in Mice.

Fig. S3. Pseudotime analysis by Monocle of CCR2^+^ Macrophages and CCR2^-^ Macrophages.

Fig. S4. Treatment of Non-Alcoholic Fatty Liver Disease Combined with Renal Fibrosis in Mice.

Fig. S5. Differential metabolites in CCR2^+^ Macrophages and their functional analysis.

Fig. S6. Integrated Transcriptomic and Metabolomic Analysis to Identify Pathway Genes.

Fig. S7. The hepatocyte-secreted protein ANGPTL8 can bind to the PIRB receptor on macrophages.

Fig. S8. Identification of ANGPTL8 and the functional Analysis of CCR2^+^PIRB^-^ Macrophages.

Fig. S9. AAV Treatment Attenuates the Development and Function of Th17 Cells in Mice.

Fig. S10. Elevated ALOX5AP Expression in LILRB2^+^ Macrophages in Renal Tissue of Patients with NAFLD and Renal Fibrosis.

**Fig. S1**


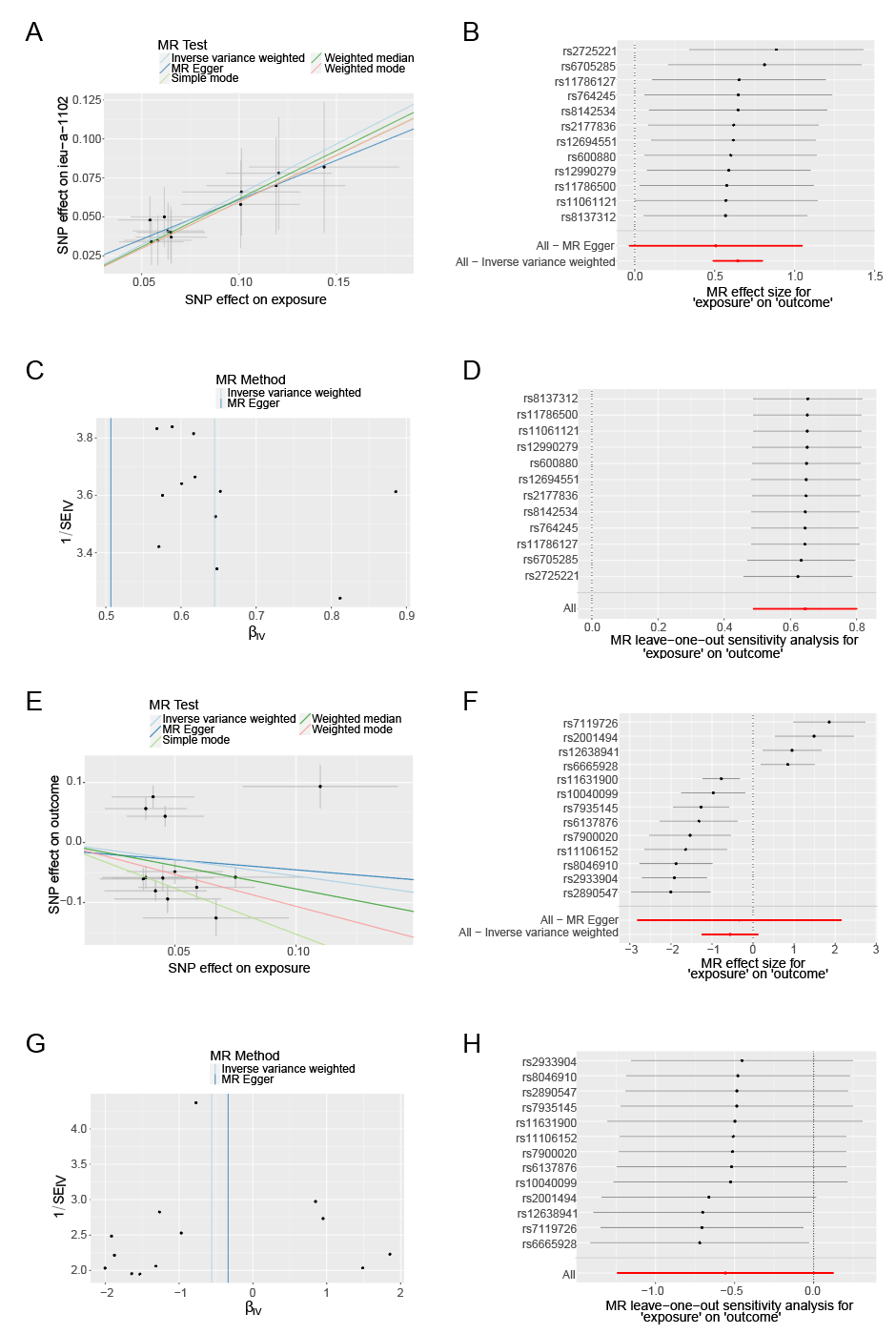


**Supplementary Figure** **S1. Results of the bidirectional MR analysis of NAFLD and CKD.**

**A**, A scatter plot estimating the effect of NAFLD on CKD. **B**, A forest plot summarizing the overall impact of NAFLD on CKD. **C**, Funnel plot of single nucleotide polymorphism (SNP) distribution in MR study of the impact of NAFLD on CKD. **D**, A sensitivity analysis conducted using a leave-one-out plot. **E**, A scatter plot estimating the effect of CKD on NAFLD. **F**, A forest plot summarizing the overall impact of CKD on NAFLD. **G**, Funnel plot of single nucleotide polymorphism (SNP) distribution in MR study of the impact of CKD on NAFLD. **H**, A sensitivity analysis conducted using a leave-one-out plot.

**Fig. S2**


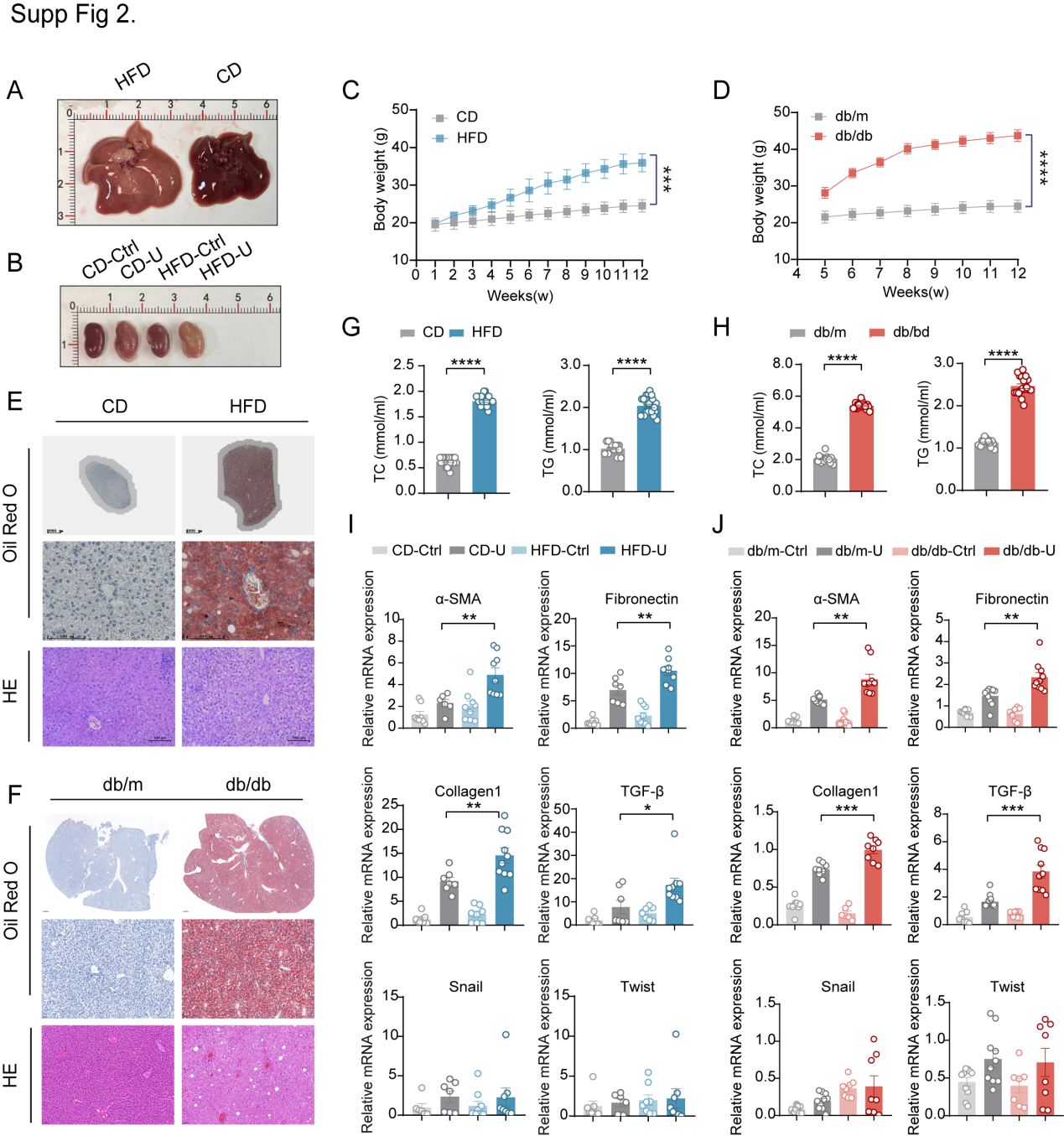


**Supplementary Figure S2. Development of NAFLD Model in Mice.**

**A**, Macroscopic images of livers from HFD Mice and CD Mice. **B**, Macroscopic images of kidneys after seven days post-UUO in HFD Mice and CD Mice, with the right kidney remaining unligated and the left kidney ligated. **C**, Weight change curves of HFD Mice and CD Mice. **D**, Weight change curves of db/db and db/m mice. **E**, Oil Red O staining and HE staining of livers from HFD Mice and CD Mice, with scale bars of 2000 μm and 100 μm, respectively. **F**, Oil Red O staining and HE staining of livers from db/db and db/m mice, with scale bars of 2000 μm and 100 μm, respectively. **G**,Plasma levels of TC and TG in HFD Mice and CD Mice. **H**,Plasma levels of TC and TG in db/db and db/m mice. **I**, RT-qPCR analysis of mRNA expression levels of fibrosis genes in kidney tissue of HFD Mice and CD Mice. **J**, RT-qPCR analysis of mRNA expression levels of fibrosis genes in kidney tissue of db/db and db/m mice **C-D, G-H, I-J**: The data are presented as the means ± SEM (n = 6-24). Group comparisons were analyzed using a two-tailed Student's T-test (black asterisks); ***p <* 0.01, ****p <* 0.001, *****p <* 0.0001.

**Fig. S3**


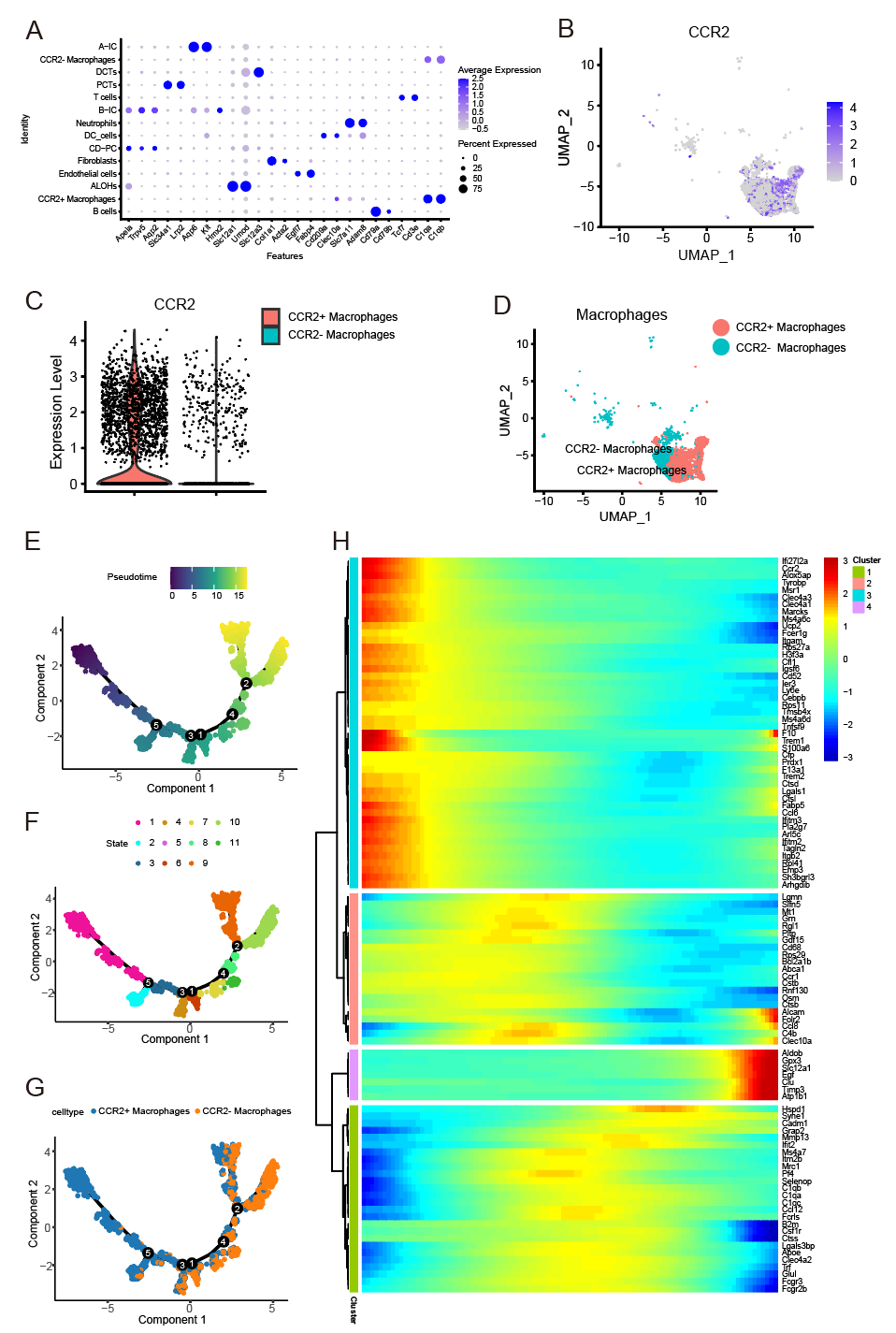


**Supplementary Figure S3. Pseudotime analysis by Monocle of CCR2^+^ Macrophages and CCR2^-^ Macrophages.**

**A**, The bubble plot shows the representative genes of 14 distinct cell types. **B**, Subclustering analysis of Macrophages in the UUO kidney. **C-D**, Violin plots and UMAP plots show the expression of CCR2 in the two subtypes. Pink indicates CCR2^+^ Macrophages, green indicates CCR2^-^ Macrophages. **E-F**, Pseudotime analysis by Monocle of Macrophages in UUO kidneys, showed the populations of macrophages in 5 branches and 9 differentiated states. **G**, Pseudotime analysis by Monocle of Macrophages in UUO kidneys, showing CCR2^+^ Macrophages and CCR2^-^ Macrophages. **H**, Genes showing change in expression along the cell differentiation trajectory by Monocle. Red indicates higher expression, blue indicate lower expression.

**Fig. S4**

**
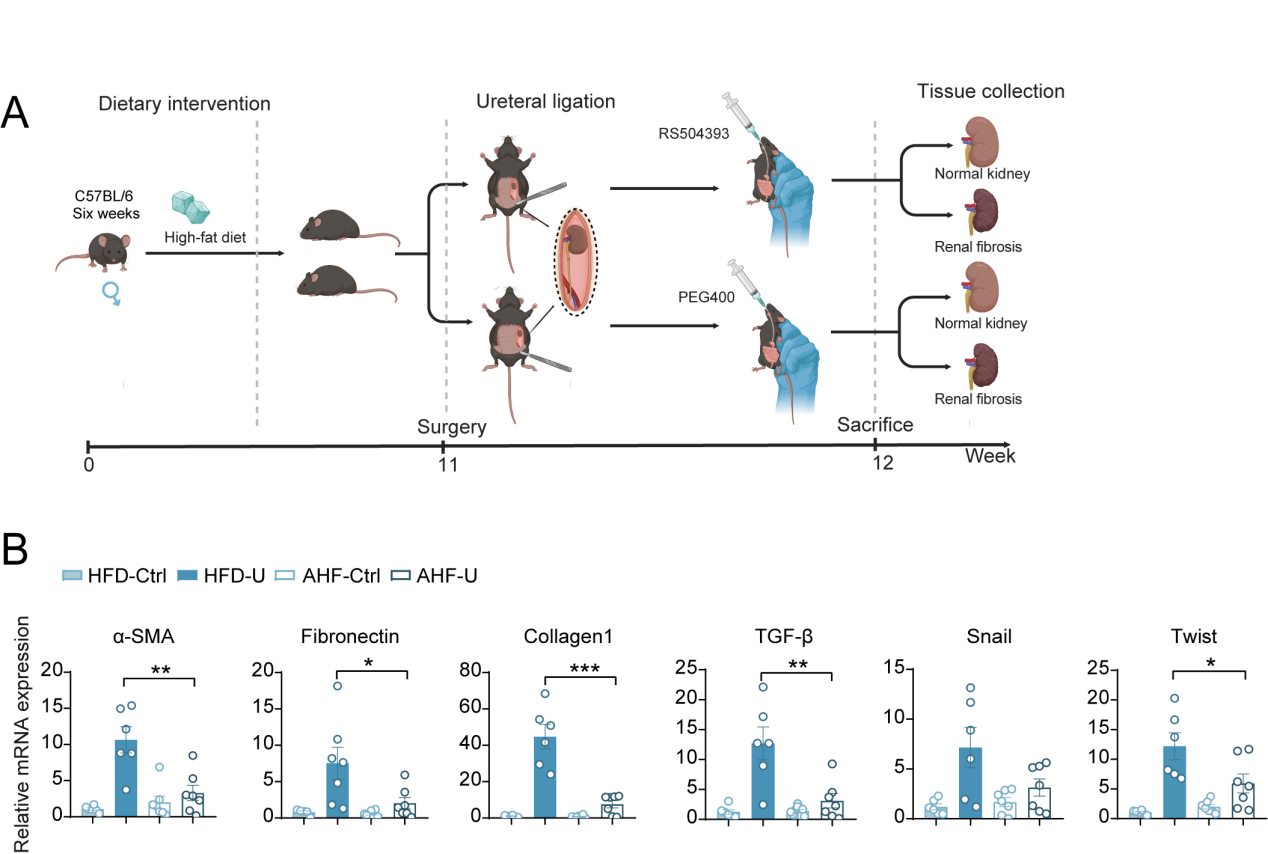
**

**Supplementary Figure S4. Treatment of Non-Alcoholic Fatty Liver Disease Combined with Renal Fibrosis in Mice.**

**A**, Treatment protocol for non-alcoholic fatty liver disease combined with renal fibrosis: Obese mice induced by a high-fat diet were divided into two groups. The experimental group was gavaged with the CCR2 inhibitor RS504393, and the control group was gavaged with PEG400. The treatment began on the day of UUO surgery and continued for seven days, with sacrifice on the seventh postoperative day for analysis of both kidneys. **B**, RT-qPCR analysis identifying mRNA expression levels of fibrosis-related genes in the renal tissues of HFD mice and treated with CCR2 inhibitor (AHF). **B**: The data are presented as the means ± SEM (n= 6-8). Group comparisons were analyzed using a two-tailed Student's T-test (black asterisks); ***p <* 0.01, ****p <* 0.001.

**Fig. S5**


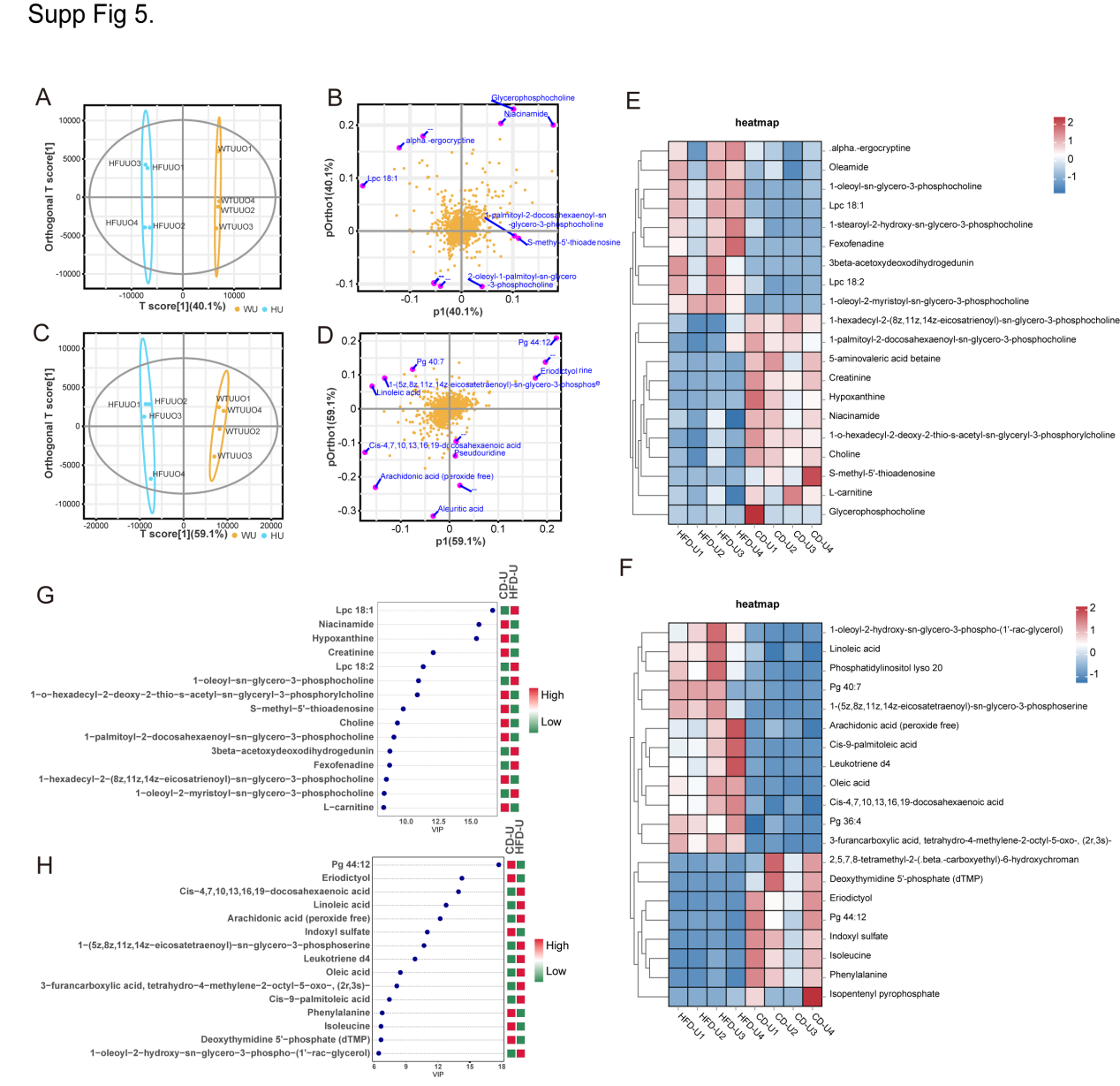


**Supplementary Figure S5. Differential metabolites in CCR2^+^ Macrophages and their functional analysis.**

**A**, Differential metabolites score plot of POS mode OPLS-DA analysis. **B**, Loadings plot of POS differential metabolites in OPLS-DA analysis. **C**, Score plot of NEG mode OPLS-DA analysis for differential metabolites. **D**, Loadings plot of NEG differential metabolites in OPLS-DA analysis. **E**, Heatmap of top 20 clustered differential metabolites in POS mode. **F**, Heatmap of top 20 clustered differential metabolites in NEG mode. **G**, VIP diagram of differential metabolites in POS.The ordinate is the differential metabolites, and the abscissa is the enrichment factor. **H**, VIP diagram of differential metabolites in NEG.

**Fig. S6**


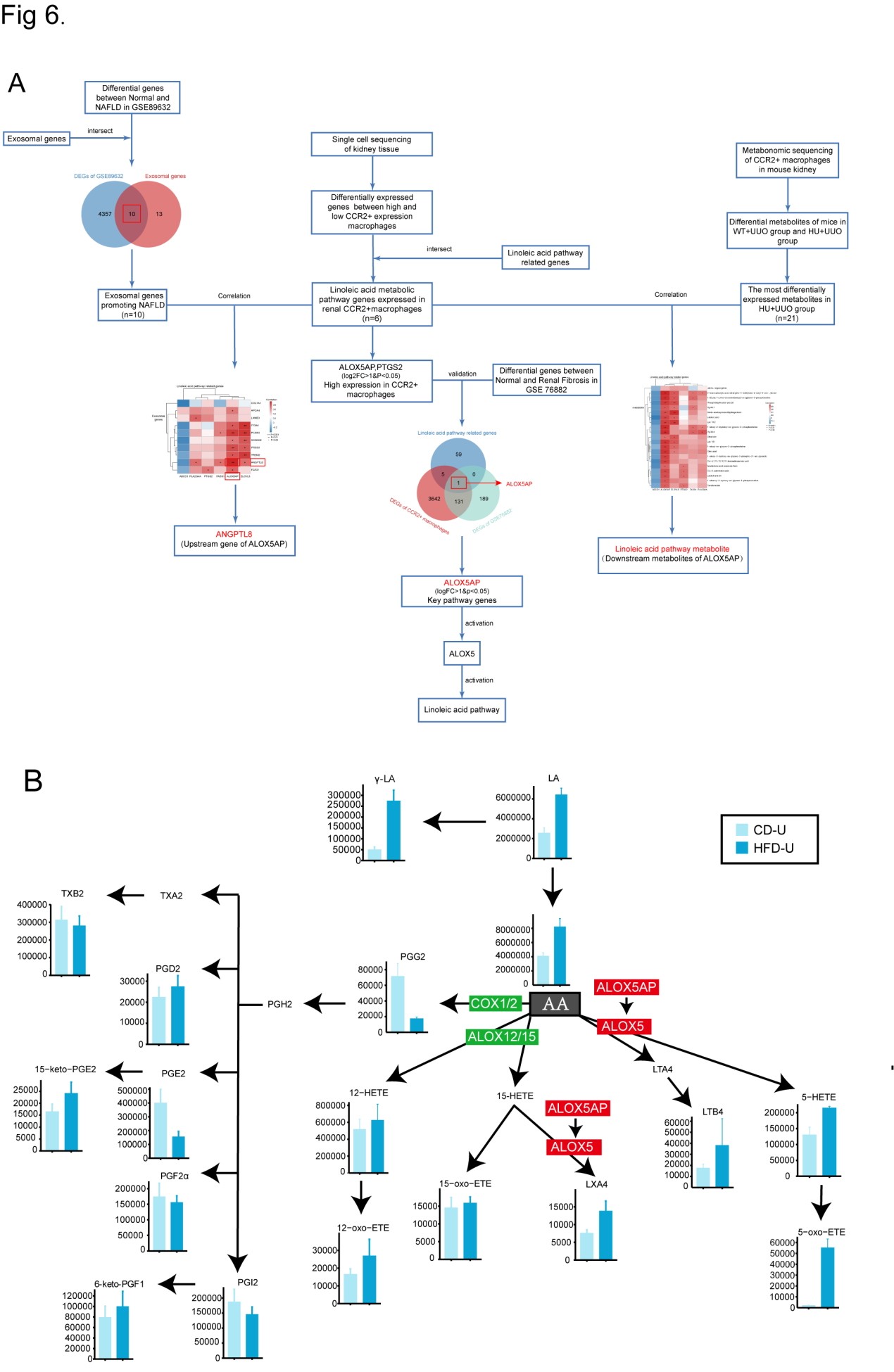


**Supplementary Figure S6. Integrated transcriptomic and metabolomic analysis to identify pathway genes.**

**A,** Flowchart of the integrated transcriptomic and untargeted metabolomic analysis process. **B,** Expression levels of linoleic acid metabolism-related metabolites in CCR2^+^ macrophages from control and high-fat diet-induced NAFLD mice 7 days after UUO.

**Fig. S7**


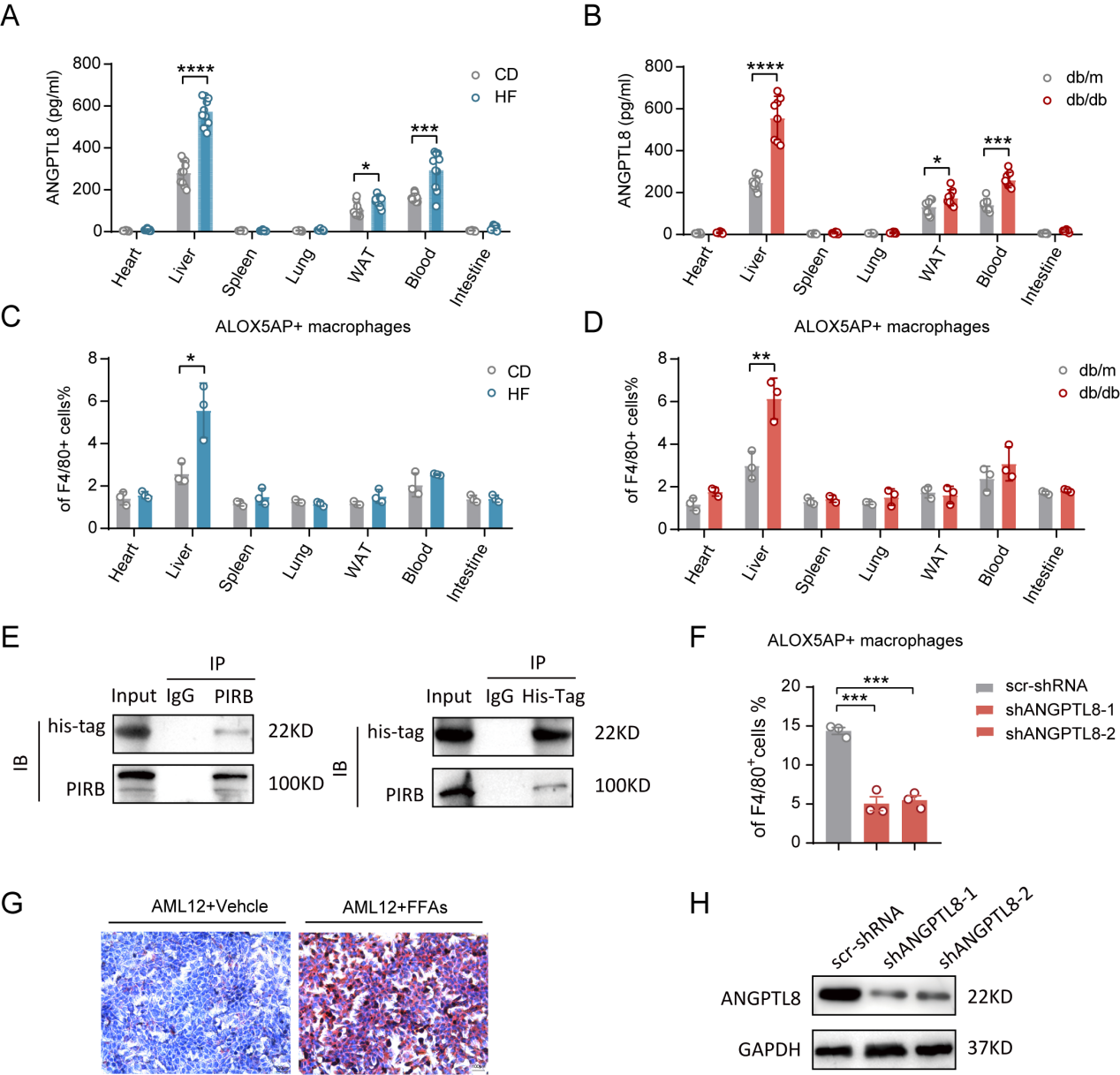


**Supplementary Figure S7. The hepatocyte-secreted protein ANGPTL8 can bind to the PIRB receptor on macrophages.**

**A,** ELISA detection of ANGPTL8 protein levels in various organs of HFD and CD mice. **B,** ELISA detection of ANGPTL8 protein levels in various organs of db/db and db/m mice. **C**, Flow cytometry analysis of the impact of secretory proteins from various organs of HFD and CD mice on BMDM cells. **D**, Flow cytometry analysis of the impact of secretory proteins from various organs of db/db and db/m mice on BMDM cells. **E**, Immunoprecipitation and Western blot analysis revealed the binding of recombinant ANGPTL8 protein(his-tag) with PIRB on BMDM cells. **F**, After ANGPTL8 knockdown in hepatocytes, co-culture with BMDM cells was performed to detect the proportion of ALOX5AP^+^ macrophages(n = 3). **G**, Representative images of lipid droplets in Steatotic AML12 mouse hepatocytes, induced and detected by Oil Red O staining. Images were captured at a magnification of 200x. **H**, Protein level analysis in Steatotic AML12 cells with ANGPTL8 knockdown. **A-D, F**: The data are presented as the means ± SEM (n = 8-10). Group comparisons were analyzed using a two-tailed Student's T-test and ANOVA with Turkey's multiple comparisons test; * p < 0.05, ** p < 0.01, *** p < 0.001, **** p < 0.0001. WAT: white adipose tissue.

**Fig. S8**


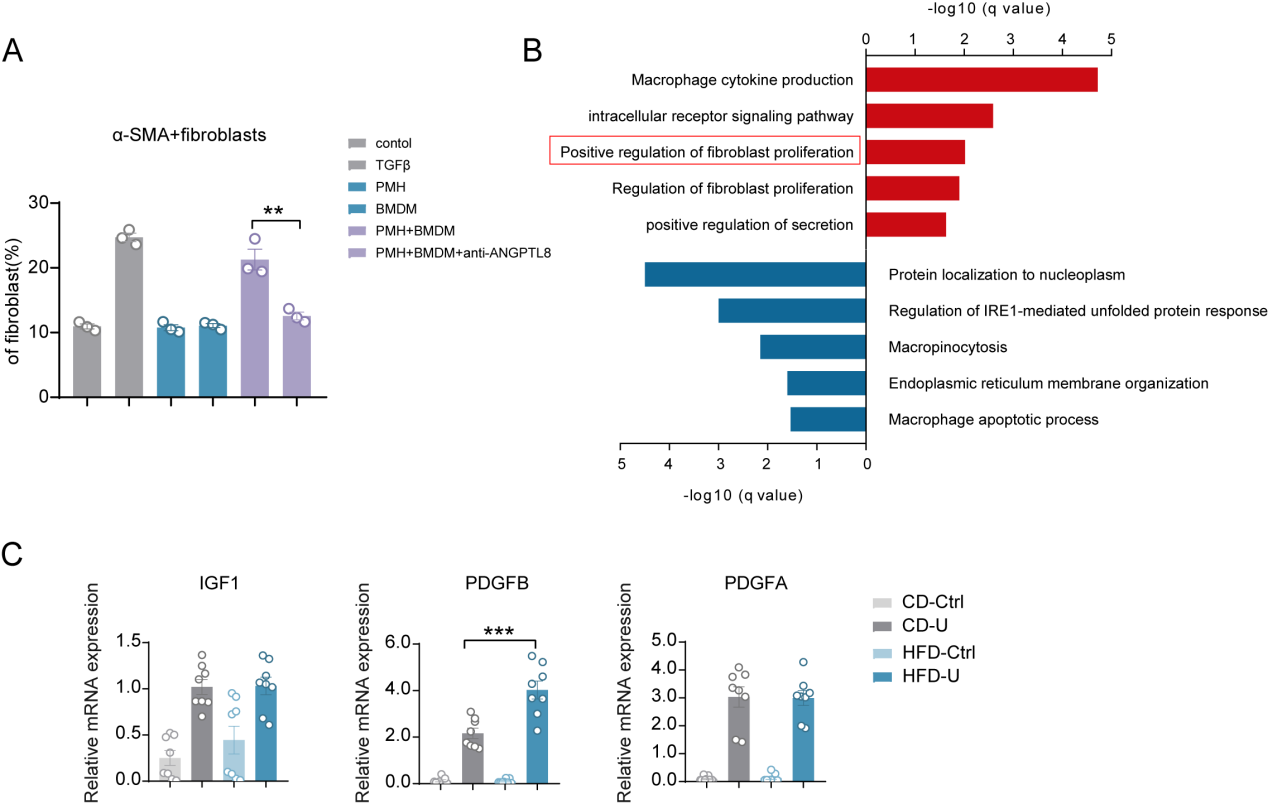


**Supplementary Figure S8. Identification of ANGPTL8 and the functional Analysis of CCR2^+^PIRB^-^ Macrophages.**

**A,** An in vitro model was established by co-culturing steatotic PMH with BMDM and fibroblasts, while simultaneously conducting experiments with ANGPTL8 neutralizing antibodies to block its activity (n = 3). **B,** KEGG enrichment analysis of differentially expressed genes in CCR2^+^PIRB^-^ macrophages. **C,** RT-qPCR measurement of mRNA levels of three exocrine function-related genes (IGF1, PDGFB, PDGFA) in the cell proliferation pathway. **A,C**: The data are presented as the means ± SEM (n = 8). Group comparisons were analyzed using ANOVA with Turkey's multiple comparisons test; ** p < 0.01, *** p < 0.001.

**Fig. S9**


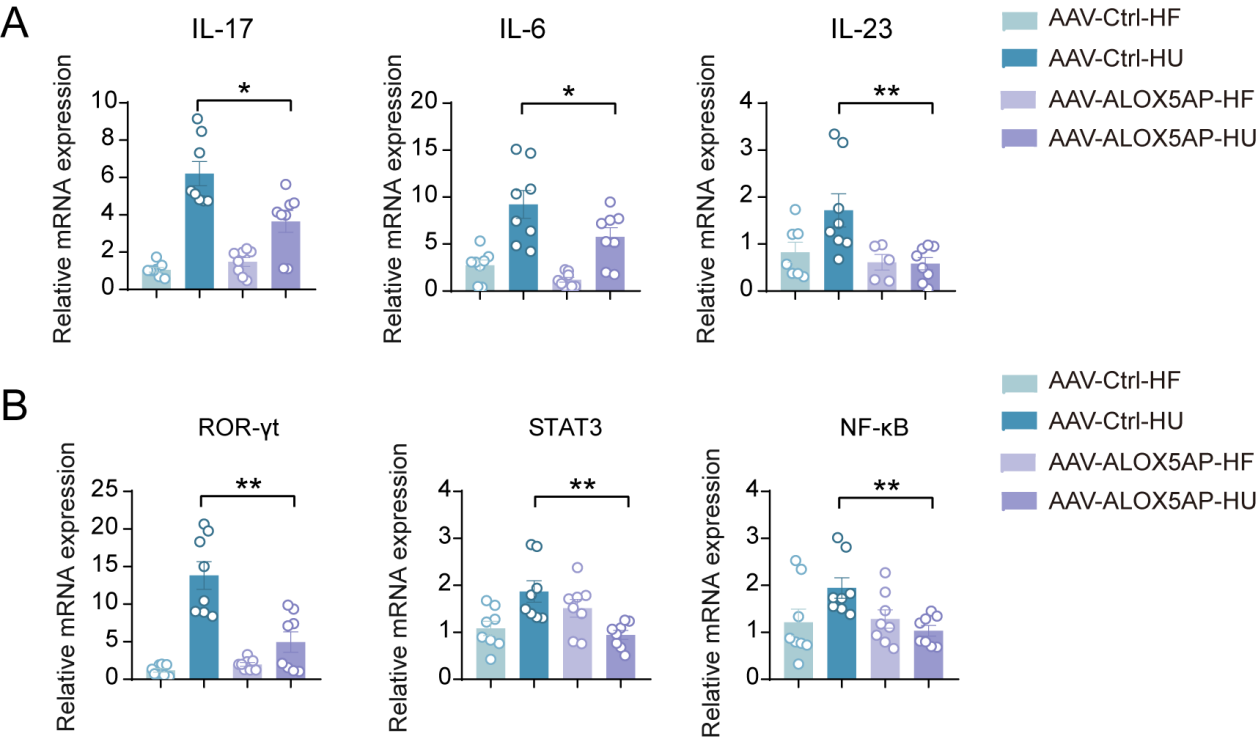


**Supplementary Figure S9. AAV Treatment Attenuates the Development and Function of Th17 Cells in Mice.**

**A,** RT-qPCR analysis of mRNA expression levels of inflammatory factors related to Th17 cell polarization in kidney tissues of the viral treatment group (AAV-ALOX5AP-HF, AAV-ALOX5AP-HU) and the control group (AAV-Ctrl-HF, AAV-Ctrl-HU). **B,** RT-qPCR analysis of mRNA expression levels of transcription factors related to Th17 cell development in kidney tissues of the viral treatment group and the control group. **A-B**: The data are presented as the means ± SEM (n = 8). Group comparisons were analyzed using a two-tailed Student's T-test ; * p < 0.05, ** p < 0.01.

**Fig. S10**


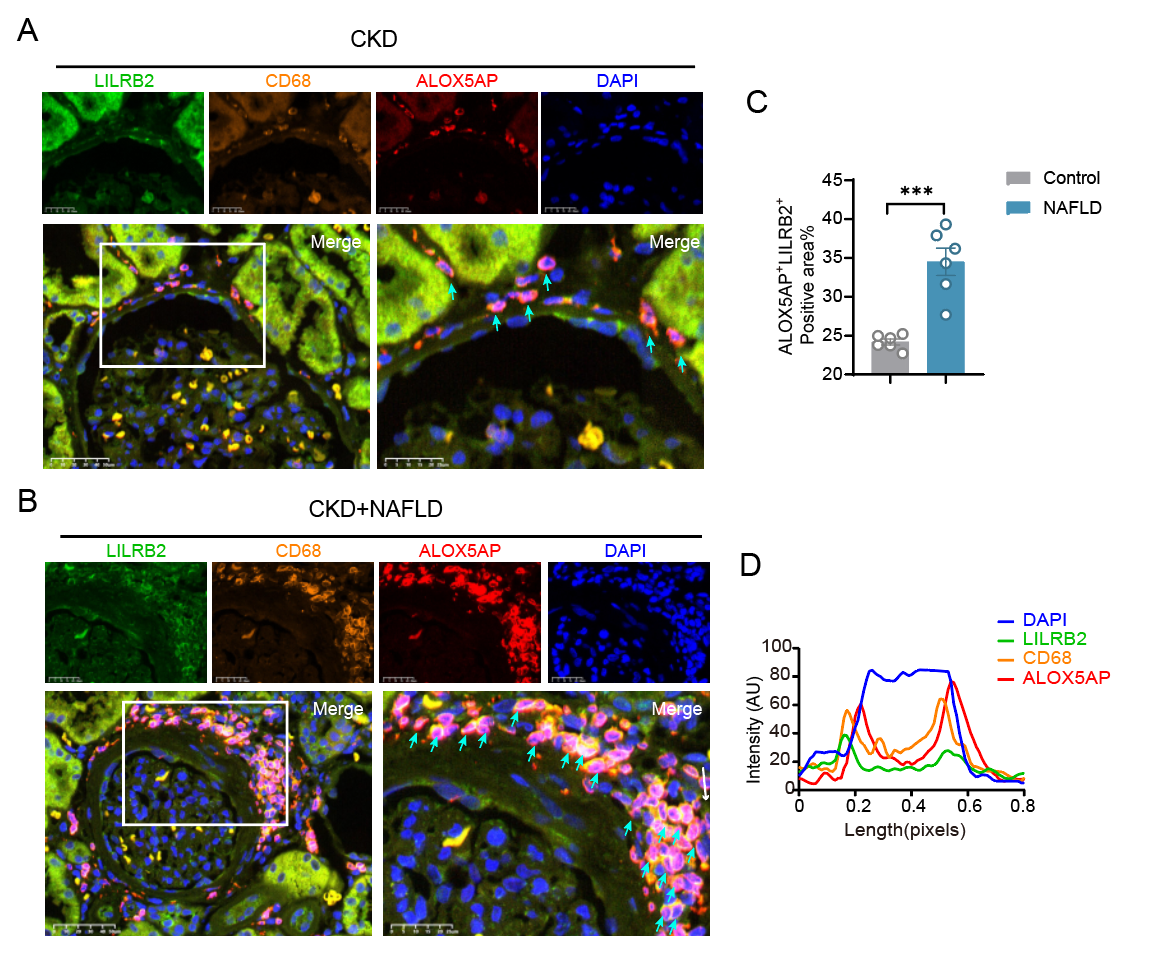


**Supplementary Figure S10. Elevated ALOX5AP Expression in LILRB2^+^ Macrophages in Renal Tissue of Patients with NAFLD and Renal Fibrosis.**

**A**, A representative renal section from a CKD patient with renal fibrosis, showing multicolor immunostaining for LILRB2, CD68, and ALOX5AP. The scale bar in the lower left image is 50 μm. The lower right image is a magnified cropped view of the white-boxed region, with the top image showing the single-channel views. The scale bar is 25 μm. **B**, A representative renal section from a CKD patient with renal fibrosis and NAFLD (CKD+NAFLD), showing multicolor immunostaining for LILRB2, CD68, and ALOX5AP. The scale bar in the lower left image is 50 μm. The lower right image is a magnified cropped view of the white-boxed region, with the top image displaying the single-channel views. The scale bar is 25 μm. **C**, Cell counts and positive area percentages for LILRB2^+^, CD68^+^, and ALOX5AP^+^ cells in tissue sections from both patient groups (n = 6). **D**, Magnified cropped image from the white-boxed region in panel B, showing intensity distribution and colocalization along the direction of the white arrow.**C**: The data are presented as the means ± SEM. Group comparisons were analyzed using a two-tailed Student's T-test ; * p < 0.05, ** p < 0.01.
